# Scaling agricultural mechanization services in smallholder farming systems: Case studies from sub-Saharan Africa, South Asia, and Latin America

**DOI:** 10.1016/j.agsy.2020.102792

**Published:** 2020-04

**Authors:** Jelle Van Loon, Lennart Woltering, Timothy J. Krupnik, Frédéric Baudron, Maria Boa, Bram Govaerts

**Affiliations:** a CIMMYT (International Maize and Wheat Improvement Center), Carretera México-Veracruz km. 45, El Batán, Texcoco, C.P. 56237, Estado de México, Mexico; b CIMMYT-Bangladesh, House 10/B. Road 53. Gulshan-2, Dhaka 1213, Bangladesh; c CIMMYT Southern Africa Regional Office, 12.5 km Peg Mazowe Road, Harare, Zimbabwe

**Keywords:** Appropriate mechanization, Service models, Enabling environment, Rural entrepreneurship, Transition framework

## Abstract

There is great untapped potential for farm mechanization to support rural development initiatives in low- and middle-income countries. As technology transfer of large machinery from high-income countries was ineffective during the 1980s and 90s, mechanization options were developed appropriate to resource poor farmers cultivating small and scattered plots. More recently, projects that aim to increase the adoption of farm machinery have tended to target service providers rather than individual farmers. This paper uses the Scaling Scan tool to assess three project case studies designed to scale different Mechanization Service Provider Models (MSPMs) in Mexico, Zimbabwe, and Bangladesh. It provides a useful framework to assess the gap between international lessons learned on scaling captured in forty tactical questions over ten “scaling ingredients” as perceived by stakeholders involved in the projects, as well as private sector actors and government employees. Although at first sight the case studies seem to successfully reach high numbers of end users, the assessment exposes issues around the sustainable and transformative nature of the interventions. These are highly influenced by the design of the projects and by the environment and context of the intervention areas. Across the three case studies, large-scale adoption of the models was found to be hampered by lack of finance to set up MSPMs and insufficient collaboration among the value chain actors to strengthen and foster Mechanization Service Provider (MSP) entrepreneurs. Applying a scaling perspective on each case study project exposed important lessons on minimizing project dependencies. Positive examples include integration of capacity development materials in vocational training centers in Zimbabwe, promotion of MSPMs by other donors in East Africa and levering of nearly USD six million of private sector investment in appropriate machinery in Bangladesh. On the other hand, there is still a high dependency on the projects in terms of coaching of service providers, facilitating collaboration along the value chain, and provision of leadership and advocacy to address issues at governance level. These results have important implications for similar development interventions aimed at increasing smallholder access to mechanization services at scale and is to our knowledge the first cross-continental assessment of these issues to date.

## Highlights

• Service providers can successfully improve access to appropriate mechanization.
• Applied a novel analytical tool to analyze constraints to scaling in three countries.
• Stakeholders are dependent on projects to address bottlenecks for scaling.
• Service providers need support to transition from project to market finance.
• Scaling Scan helps stakeholders identify opportunities to accelerate scaling.

## 1 Introduction

### 1.1 Agricultural mechanization as a vehicle for positive rural transformation

Agricultural mechanization is the continued introduction of farm equipment to make activities such as land preparation, crop production, harvesting, processing, and transport of goods more efficient. Increases in resource use efficiency, as well as labor and land productivity, are achieved through higher precision and timely operations, reduced drudgery and overall savings in labor, leading to higher quality of life (Clarke, 2000; Clarke and Bishop, 2002; Reid, 2011; Sims and Kienzle, 2016). Mechanization is regarded as a motor for agricultural transformation; only very specific farm operations are still accomplished manually in high- and middle- income countries (Schmitz and Moss, 2015), while farm machinery and tractor power in sub-Saharan Africa, parts of Latin America, and South Asia is almost negligible given total cultivated land area (Baudron et al., 2015; Mrema et al., 2008).

Yet, the advantages of mechanization options that are appropriate for the field sizes and resource endowments of smallholder farmers can be considerable (Sims and Kienzle, 2016). This is especially the case in countries with growing manual labor shortages caused by the rural to urban migration of youth, what in turn increases pressure on “those left behind”, especially for female headed farm households (Baudron et al., 2015; Rosegrant et al., 2014; Sims and Kienzle, 2017). Despite increasing interest by policymakers and international donors to use mechanization as a vehicle for effective rural transformation (FAO, 2008; Mrema et al., 2014), efforts to improve mechanization of smallholder agriculture have not been consistently successful.

### 1.2 Challenges for smallholders to access mechanization

The most common challenges to increasing smallholder access to appropriate mechanization include (1) a mismatch between the economies of scale of machines and farm size. More than 50% of globally produced food originates from small family farms (Herrero et al., 2017), and many of these consist of separate and dispersed fields and thus are poorly suited for larger machinery (Harman, 2016; Krupnik et al., 2013; Maass-Wolfenson, 2013). (2) Cost of machinery. In many countries, farmers cannot afford to purchase equipment and financial support through subsidies or finance schemes are limited. The financial-service sector shies away from providing credit to smallholders because of lack of eligible collateral and a perception of high risk involved with agriculture (Clarke, 2000; Holtkamp and Lorenz, 1990; Mottaleb et al., 2016; Mrema et al., 2008; Sims et al., 2016). (3) Technocratic attempts to “leapfrog” the agricultural mechanization process and introduce machinery without addressing farmers' capacity and educational needs undermines the process to establish skilled people able to operate, maintain, and repair equipment (Diao et al., 2018). (4) Many rural mechanization initiatives in developing countries are centrally planned and as they directly target farmer cooperatives as major beneficiaries often fail to increase access for smallholders due to insufficient support of complementary actors involved with manufacturing, supply, distribution, sales, and after-sales services (Clarke, 2000; FAO, 2008; Pingali et al., 1988).

### 1.3 Appropriate mechanization

**Fig. 1:**
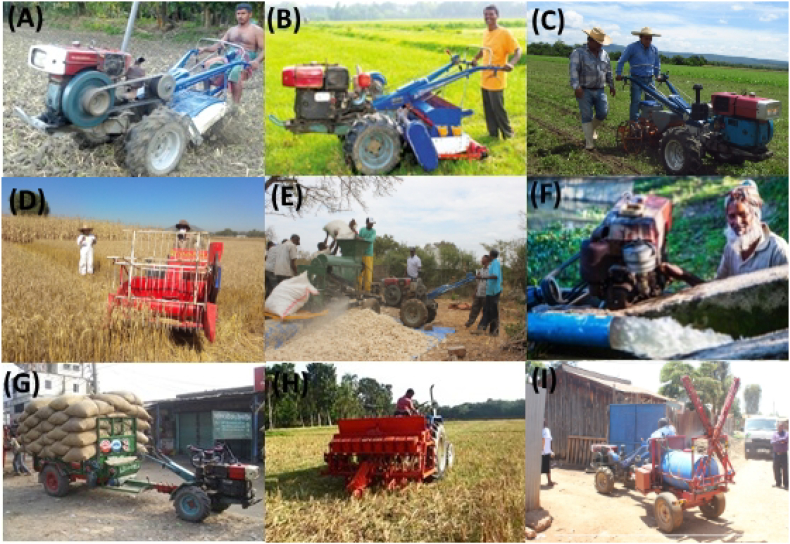
Selected “appropriate” agricultural machineries used in the context of smallholder agriculture: (A) two-wheeled tractor and the removable power tiller, (B) two-wheeled tractor driven direct seed and fertilizer drill, (C) two-wheeled tractor single row planter, (D) self-propelled rice and wheat reaper, (E) two-wheeled tractor propelled stationary maize sheller, (F) axial flow pump powered by a two-wheeled tractor, (G) two-wheeled tractor trailer used to haul agricultural produce to the market, (H) “Happy Seeder” used to drill seed into rice residue without tillage, (I) small-scale sprayer cart (Photos: T. Krupnik, Md. A. Matin, S. Justice, R. Martin, A. Haque, F. Baudron, J. López).

The development and testing of “appropriate” mechanization options for smallholder farmers address the first three challenges listed above. Appropriate refers to user-friendly machinery tailored to smallholders' fields and suitable to local agronomic circumstances and limited resource endowments (Krupnik et al., 2013; Mottaleb et al., 2016; Van Loon et al., 2018). The technologies are designed to have minimal negative social and environmental consequences. Fig. 1 shows some examples of such equipment. Rather than consolidating small farms to suit large machines, scale-appropriate machinery is adapted to farm size and production conditions. For example, two-wheeled tractors are relatively affordable and easily maneuverable in small-sloped lands with presence of trees in the field. These machines, adopted at large scale, have advantages over four-wheeled tractors and animal traction in terms of operating costs (Baudron et al., 2015; Lara-Lopez and Chancellor, 1999), lower emissions (Baudron et al., 2015; Tubiello et al., 2015) and less soil compaction due to their lower weight. Additionally, appropriate mechanization has less impact on social structures as farmers can easily revert to animal traction or manual labor because small machinery does not require significant land consolidation arrangements and investment is significantly less when compared to larger-level machinery. As such, appropriate mechanization is part of a sustainable framework for improved efforts in rural farm mechanization (Baudron et al., 2015; López Gómez and Van Loon, 2018; Sims and Kienzle, 2017).

### 1.4 Service models to access appropriate mechanization

Despite the small scale and increased affordability of many appropriate mechanization options increasing smallholder access to machinery on a large scale remains challenging. However, smallholders can benefit from the use of machinery through low-cost rental or service providers and hiring arrangements that reduce farmers' individual cost burdens of purchasing, owning, and maintaining machines (Diao et al., 2018; Mrema et al., 2014; Sims and Kienzle, 2017). Nonetheless these options can come with increased transaction costs (Laxmi et al., 2007) that require offsets and appropriate accounting for in the respective business models. Further, service provider arrangements can enable farmers who own and operate machines to become rural entrepreneurs by using machinery for remunerative on- and off-farm activities (Sims et al., 2018). Such service bundling can assist in more rapid recovery of machinery investments by offering a diversified set of services to farmer-clients (Baudron et al., 2015). Where rural-to-urban and international (e)migration occurs due to farmers seeking more remunerative employment options, machine service provides a buffer against increasing labor costs and scarcity in rural economies (Gartaula et al., 2012; Biggs and Justice, 2015).

### 1.5 Scaling of innovations

Over the last five years, development organizations have started to look more seriously at scaling: how a successful transition from initial farmer adoption in pilot projects to self-propelling and sustained uptake of technologies can be implemented more systematically (Chandy et al., 2013; Cooley and Linn, 2014). Woltering et al. (2019) state that meaningful impact at scale rarely occurs within a project lifetime or context, but emerges when new ways of working are becoming accepted by a critical mass of actors in society. Scaling draws on the notion that technology adoption relies largely on parallel and supporting innovations in other sectors such as finance, public governance, and capacities. Scaling frameworks (Cooley, 2016; Jacobs et al., 2018; Kohl and Foy, 2018) help improve understanding about the aggregate influence of these innovations and how they foster an environment enabling adoption.

### 1.6 Scope of the paper

In this paper, we focus on three case studies in which the International Maize and Wheat Improvement Center (CIMMYT) and partner organizations aim to improve farmers access to appropriate agricultural mechanization through different Mechanization Service Provider Models (MSPMs): (1) the Machine Hire Centers (MHCs) supported by *MasAgro* and aligned projects in Mexico, (2) the Zimbabwean experience from the Farm Mechanization and Conservation Agriculture for Sustainable Intensification (FACASI) project, and (3) the Cereal Systems Initiative for South Asia – Mechanization and Irrigation (CSISA-MI) project in Bangladesh. The paper assesses the extent to which each initiative fits with the needs of the environment to enable sustained use of machinery by farmers at a large scale and acknowledges the influence of project design on the outcomes. As such, the paper provides an insight on how a common problem is approached from different starting points and how apparent success at an intermediate, on-going point in a project's lifetime can be assessed to identify critical aspects of needed exit strategies.

## 2 Materials and methods

### 2.1 Case study descriptions

The three case study regions and the models used for engagement with Mechanization Service Providers (MSPs) are described in Table 1.

**Table 1:** Description of the Mechanization Service Provider Models as utilized in the MasAgro initiative, the FACASI project, and the CSISA-MI project in Mexico, Zimbabwe, and Bangladesh, respectively.

	*MasAgro* (Mexico)	FACASI (Zimbabwe)	CSISA-MI (Bangladesh, Phase I)
Project or program context	• Component within *MasAgro* program (2009–2019) and part of a Government public policy framework	• FACASI (2014–2019)	• CSISA-MI (2013–2018, first phase) emerged from the broader CSISA program (2009–2020)
CIMMYT primary partner(s)	• Government for funding (the Ministry of Agriculture (SADER, or Secretary of Agriculture and Rural Development, formerly SAGARPA Secretary of Agriculture, Livestock, Rural Development, Fisheries and Food) under the Government of Mexico) • Multi-stakeholder operation of the project including public and private sector, research and extension.	• University of Zimbabwe, vocational training centers, local manufacturers	• International Development Enterprises (iDE), Bangladesh Agricultural Research Institute (BARI), Department of Agricultural Extension (DAE), and a range of private sector partners.
Types of individuals targeted to become service providers	• Farmers association representatives, farm advisors, rural entrepreneurs	• Medium-sized farmers, rural entrepreneurs	• Individual farmers with experience and/or ownership of two-wheel tractors or multipurpose engines
Targeted farmer end-users	• Small to medium scale cereal-based farmers (~0–20 ha)	• Smallholders farmers (~0–10 ha)	• Smallholder farmers (< 1 ha on average)
Initial selection criteria for service providers	• Minimum infrastructure, logistics capability, initial client base	• Initial purchase power, no initial infrastructure or equipment required, no client base	• Initial purchase power, credit worthiness, potential to serve smallholder farmer clients
Perceived objective of service providers	• Complementary income from renting out machine and associated services	• Machine services as complementary income for existing service providers	• Machine services as a professional business
Main machine package	• Accessories for four-wheeled and two-wheeled tractors, animal-drawn and manual equipment including seed drills, fertilizer, and land preparation equipment	• Two-wheel tractors and accessories and/or shellers of various sizes	• Two-wheel tractor seed and fertilizer drill attachments, attachable and self-propelled reapers, axial and mixed flow irrigation pumps, small multi-crop reaper-binders and combines
Ownership of machines by service providers	• Machines are provided and owned by *MasAgro* without direct ownership by service providers	• Yes (individual or group), pay 10% and remaining 40–90% over 3 years	• Yes (individual service providers)

#### 2.1.1 MasAgro's machine hire centers in Mexico

In Latin America, the core activities on mechanization take place within the *MasAgro* initiative supported by the Government of Mexico through the Ministry of Agriculture (SADER or Secretary of Agriculture and Rural Development, formerly SAGARPA Secretary of Agriculture, Livestock, Rural Development, Fisheries and Food). *MasAgro* aims to increase resilience of maize and wheat agri-food systems, primarily in rain-fed conditions through the adoption of sustainable agricultural practices (Romero-Perezgrovas et al., 2014), and by stimulating strategic alliances in the value chain works to optimize the agri-food systems through iterative and participatory discussions. Inherent to *MasAgro* is a de-centralized network of local research partners, extension agents (including the national agricultural research and education system (NARES)) and farmer collaborators -coined innovation hubs- embedded within small- and medium-sized farm communities throughout Mexico. These hubs are developed to facilitate feedback on farmers' needs, to adequately address local conditions, and to provide a platform to reach out to and interact with relevant actors of the value chain (i.e., farm advisors, local manufacturers, service providers, and farm input distributors) (Camacho-Villa et al., 2016; IICA, 2016; Liedtka et al., 2017). Through the *MasAgro* initiative as a governmental supported country intervention, machines and technical assistance are provided to farmers as a means to improve farming practices, and as a pathway to make innovative technology accessible and catalyze awareness. The development of Machine Hire Centers (MHCs) provides access to appropriate farm mechanization options. CIMMYT identifies farmer association representatives, farm advisors, local NARES representatives, or alternative businesses like production integrators who provide paid machine services as a component of its overall farmer support activities. They qualify to become MHCs when they can demonstrate logistic capability (ability to maintain and operate machinery), infrastructure (to house machinery and repair equipment), and proven ability to form an initial farmer-client base. The primary approach is not to push farmers to use or provide free of charge availability of project-owned machinery, but to stimulate the notion of entrepreneurship in rural communities. Furthermore, they formally agree to provide space for farmers' and farm advisors' educational meetings on equipment and improved agronomy principles, participation in and maintenance of research and demonstration plots, and day-to-day administration, registration, and financial management of performed service activities. Consequently, strategic agreements with service provider candidates are made to integrate end-users in a functional value chain. In return, the *MasAgro* initiative provides them with selected farm implements at free disposal under yearly revised agreements on the condition that the implements are safeguarded and preserved in good conditions. They generate income by renting out the machinery to farmers. If the MHCs do not fulfill any of these requirements, the contract between them and CIMMYT is broken and they return the machines for reallocation. The hypothesis is that alleviating the high investment costs for machinery (with average costs of approximately USD 15,000) will bridge a first learning phase for the MHC while solidifying a potential client base and this structure facilitates the testing of several business models and interaction with supporting local extension partners, both essential to setup profitable business cases around MSPMs. In this sense, a certain degree of dis-adoption is expected as part of the selection and optimized targeting process of setting up MSPs in a new locality as this ‘tester’ phase (Kiptot et al., 2007) allows for administrative flexibility and feedback for equipment selection.

#### 2.1.2 FACASI's complementary income service providers in Zimbabwe

The second case study is FACASI's mechanization service provider efforts in Zimbabwe, although the project is active in East Africa (i.e., Ethiopia, Kenya and Tanzania) as well. The project uses a “Making Markets Work for the Poor (M4P)” approach (The Springfield Center, 2015) that aims to strengthen the supporting functions of the multiple types of markets necessary to stimulate small-scale farm mechanization, with a primary focus on two-wheeled tractor attachable implements, as well as shellers of various sizes. Implicit in this work are activities that encourage machinery demand creation, knowledge and skills development, information deployment, and access to finance. Demand creation among smallholder farmers is facilitated largely through machinery demonstrations and field days during which participants learn from farmers who utilize appropriate machinery. Knowledge and skills of service providers, but also mechanics, artisans, and manufacturers, are also developed through technical and business trainings facilitated by FACASI. The project develops the capacity of existing vocational training centers to provide ongoing machinery trainings, by supplying them with training materials and co-developing curricula. The project provides information (e.g., performance of different machines, cost-benefit analyses of mechanized farm operations, profile of farm machinery adopters) to private sector partners, based on their own demand, as “business intelligence” to expand markets. The same information is shared with development NGOs, extension services, and policymakers, and through the organization of frequent multi-stakeholder roundtables, coordination among different actors involved in small farm mechanization markets is strengthened. Finally, aspiring service providers are connected to financial institutions that provide loans for machinery purchase. In this context, mostly rural entrepreneurs and medium-sized farmers aiming to diversify their income and/or complement their farm earnings are targeted to enter into business as local MSPs. All service providers pay the minimum for starting equipment (i.e., two-wheeled tractors and accessories); however, when technology is introduced in a new area, FACASI may subsidize purchases by 50% for initial pioneering adopters.

#### 2.1.3 Expanding farmer access to appropriate mechanization through service providers and public-private sector engagement in Bangladesh

CIMMYT and the NGO International Development Enterprises (iDE) lead the first phase of the CSISA-MI project, emerging from the broader CSISA program that spans India, Bangladesh, and Nepal. The project involves contributions from the Bangladesh Agricultural Research Institute (BARI), Department of Agricultural Extension (DAE), and a range of private sector companies. The project aims to sustainably intensify agricultural systems in Bangladesh by encouraging the use of surface water irrigation and efficient agricultural machinery with support to local service providers to scale out farmers' access to two-wheeled tractor attachable equipment. Importantly, two-wheeled tractors and Chinese-made engines are already common in Bangladesh, providing an entry point for innovative equipment such as seeders, irrigation pumps, and reapers that can be attached to or driven by two-wheeled tractor engines (Krupnik et al., 2013; Mottaleb et al., 2016). The project works by developing joint venture agreements with machinery importers, manufacturers, dealers, and financial credit providers contingent on the private sector investing in machinery sales to emerging service providers. Technical support is provided by the project, but no direct financial resources are given to service providers.

**Fig. 2:**
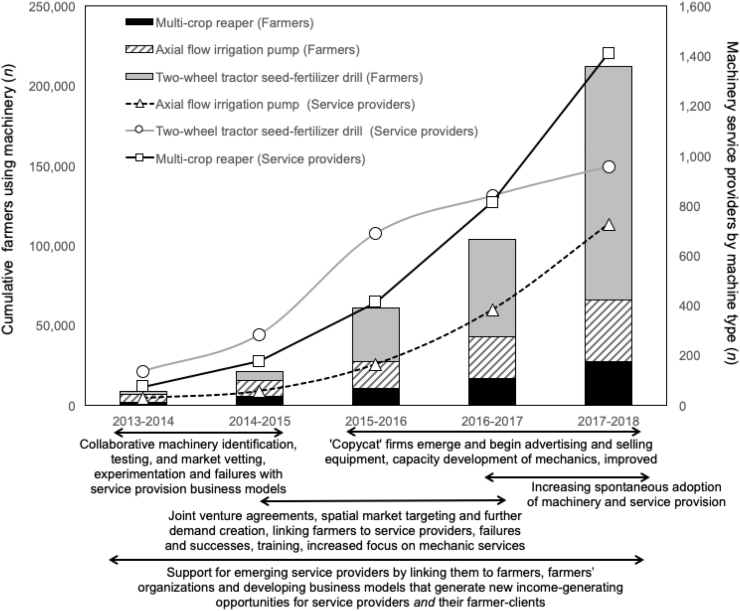
Growth in number of farmers accessing appropriate machinery (left Y-axis) through service providers (right Y-axis) in southern Bangladesh (2013–2018) and associated activities that encouraged use of machinery (adapted from McHugh et al. (2019)).

CSISA-MI uses the following mechanisms to propel public-private engagement to support the commercial availability of equipment and the expansion of service provider businesses: (A) collaborative machinery identification, testing, and market vetting, (B) setup of business partnerships to encourage private sector leadership and investment in machinery markets, (C) spatial and market targeting to facilitate demand creation, training, and awareness building of appropriate machinery, (D) capacity development of mechanics to repair and source spare parts for appropriate farm equipment and assurance of after-sales services and technical advice deployed through state extension partners, and (E) support for emerging service providers by linking them to farmers and farmers' organizations, and developing business models that generate new income-generating opportunities (Fig. 2).

### 2.2 Mechanization service provider models' scaling assessment: The “scaling scan”

We utilized the Scaling Scan (Jacobs et al., 2018) as a framework to better understand the enabling environment required for adoption of MSPMs. At the core of the Scaling Scan is a scalability assessment tool that integrates ten scaling “ingredients” (e.g., “Finance”, “Collaboration”, and “Awareness & Demand”, see Table 2), each reflecting particular sets of professional activities. Ubels and Jacobs (2016) found these ingredients to be critical for successful scaling based on interviews with experts. In contrast to other scaling frameworks that dive deep into the attributes of the innovation itself (Rogers, 2003), nine out of ten scaling ingredients focus on the non-technological conditions that determine if the system around the innovation is “scale-friendly” (Woltering et al., 2019). The Scaling Scan is an easy-to-use and readily available (in Spanish and English) tool that allows a quick and structured feedback from local stakeholders to issues that matter in scaling. Each ingredient is evaluated by four tactical questions (see Supplementary Material 1) that reflect critical international lessons learned on scaling and guide the tool's users to assess the gap with the reality on the ground. The Scaling Scan is semi-quantitative in nature, as users rate each question and end up with an average indicator score per ingredient from 1 (poor status) to 5 (very conducive to scaling), which allows them to identify which scaling ingredients represent a bottleneck for scaling. In 2018 only, at least 552 users in 10 countries were recorded to apply the Scaling Scan (Camacho-Villa, Personal Communication 2018 (unpublished data); McHugh et al., 2019). Although scoring facilitates comparisons, the true value is in identifying specific questions that generate attention and make project teams realize they may not address potential leverage points that could lead to breakthroughs in scaling. Therefore, there should always be ample time for discussion on the motivations behind the scoring. Scaling often calls for large changes that may have wide implications for society and the environment, both positive and negative (IDRC, 2018; Wigboldus, 2018). For this, the Scaling Scan includes a “responsibility check” that challenges users to assess the risk associated with reaching the scaling ambition within and beyond the geographic, social, and time boundaries set by the project. It is a check on the long-term implications on, for example, social inclusion and the environment, if the innovation indeed reaches the intended scale.

**Table 2:** Scaling “ingredients” assessed in the Scaling Scan tool and their justification (adapted from Jacobs et al. (2018)).

Ingredient name	Scaling farm mechanization service providers
Technology/practice	The Mechanization Service Provider Model should be relevant, compatible, easy to adopt, and better than alternatives that address the problem of the target population.
Awareness & demand	Farmers, service providers, and machinery companies should be aware of the technologies and service provider arrangements and demand their use.
Business cases	Attractive financial/economic propositions for companies, service providers, and other actors should be in place to respond to the demand for mechanization service providers.
Value chain	Effective links between value actors should exist for them to pursue the business cases for mechanization and service providers.
Finance	Effective and low-risk financing options for users and other value chain actors should be available.
Knowledge & skills	Individual- and institutional-level capacity should be sufficient to use, adapt, and promote the innovation.
Collaboration	Strategic collaboration within and beyond the sector is required to scale machinery and service provider businesses beyond the project context.
Evidence & learning	Evidence and facts (data, scientific insights) are available to underpin and help gain support for the pursuit of the scaling ambition.
Leadership & management	Effective coordination and navigation of the scaling process by machinery and service provider “champions” and brokers help propel scaling forward.
Public sector governance	Government support and/or lack of prohibitive policies are necessary to achieve the scaling ambition.

In this paper, we use the Scaling Scan to assess the status of three MSPMs in the case studies versus critical lessons learned for scaling. Per case study region, workshops were held in which project partners from three different sectors (i.e., 1) regional governmental representatives, 2) national and local private sector stakeholders and 3) direct project collaborators, including extension agents and site managers) relevant to mechanization at country level were selected to answer the tactical Scaling Scan questions. Participants were purposefully identified to assure the provision of insightful information from partners with experience of the case study projects, and to avoid technical language barriers. The ultimate end-users, the smallholder farmers, and target group, the MSPs, were not included as a sample group in this study, because it would require multiple translations and an adaptation of the questions to the (multiple) local contexts and specific innovation. The same researcher, with no direct involvement in the individual projects, facilitated the workshops that took about 3 h each. Before the workshops, project leaders and one of the developers of the Scaling Scan went through each step of the tool in detail and their answers were used as a reference benchmark. Forty people (in addition to the project leaders) answered the survey, aiming to have in each case study at least three persons per sample group. In Zimbabwe, participants were hesitant to answer the Scaling Scan for a general MSPM – as scores were very different depending on machinery technology - and rather opted to evaluate mechanization service businesses that either offered planting or shelling services. As such, we present results for both service types in this case study and analyze them as individual MSPM scaling scans.

The scoring per ingredient in the Scaling Scan is analyzed by taking the average score of the four corresponding tactical questions, building an indicator score for that ingredient. Fluctuations in resulting indicator scores across countries and participants are expected, but the objective of the Scaling Scan is to reveal the critical issues that need to be dealt with in order to induce a favorable scaling environment. As such, the focus in this work lies in understanding overall tendencies and address motivations between different perceptions among sample groups in the case studies. For this purpose, the use of descriptive statistics should suffice. Nevertheless, in Supplementary material 2 a quantitative approach is used to analyze the available data and backstop the descriptive indications.

## 3 Results and discussion

### 3.1 Cross-regional scaling assessment

**Fig. 3:**
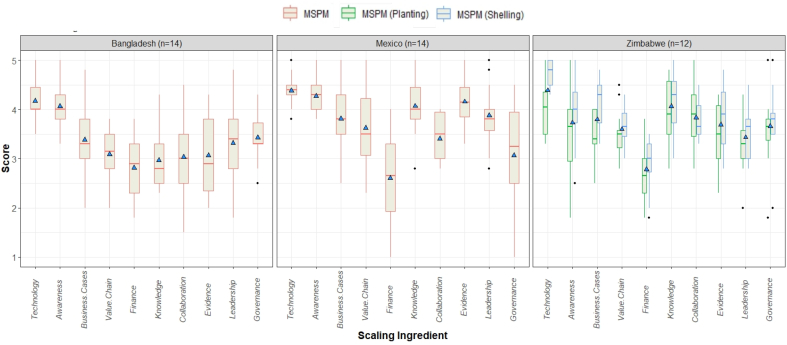
Country level scaling assessment workshop results with average scoring on the 4 tactical questions corresponding each scaling ingredient – Bangladesh (left) and Mexico (middle) with 14 participants assessing the general MSPM concept (red boxplots) within the corresponding projects' framework, and Zimbabwe with 12 participants reflecting on the MSPM with a focus on planting (green boxplots) or shelling (blue boxplots) service provision, the blue triangle gives overall average value per ingredient (score of 1 indicates a poor status while a score of 5 indicates that the particular ingredient is very conducive to scaling). (For interpretation of the references to colour in this figure legend, the reader is referred to the web version of this article.)

Fig. 3 shows the average indicator score and its spread for each scaling ingredient for the three case study countries as collected during the workshops. For the individual scores we refer to the heat maps and data set in Supplementary material 3. It can be seen that the Technology/Practice and the Awareness & Demand ingredients score high in all cases (above 4 and 3.5 respectively), Finance scores consistently low and the most contrasting opinions were found on Public Sector Governance and Leadership & Management as being conducive, or not, for scaling.

In general, participants in Bangladesh appear to be more skeptical with slightly lower resulting averages for all other ingredients, while participants in Mexico appear to have opinions that are more divergent as indicated by the larger spread of the results per scaling ingredient. In Zimbabwe, results follow the same tendency on average, and when using a one-way ANOVA to compare scoring between MSPM Planting and MSPM Shelling, the latter was rated significantly higher only for Technology and Business Cases (*t*-test: *p* < .05). This confirms that the specific technology choice of the services offered indeed influence scoring results, but for scaling an MSPM as an organizational innovation in Zimbabwe, other non-technological ingredients appear to overshadow this aspect.

**Fig. 4:**
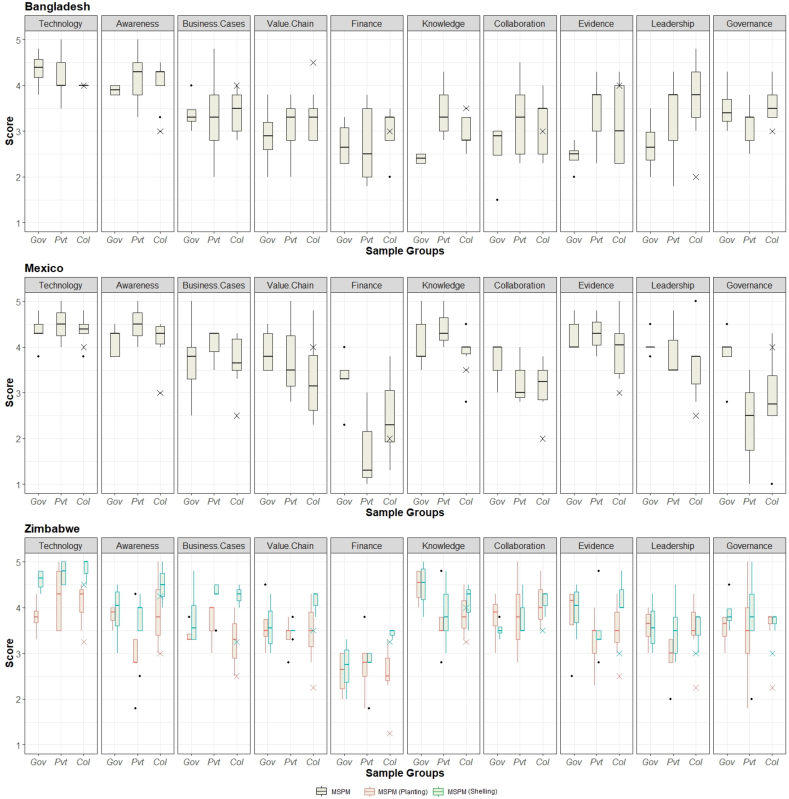
Boxplots with average scaling ingredient indicator scores per sample group (government actors - Gov, private sector – Pvt., project collaborators – Col) in all 3 case studies – black boxplots represent the results for the general MSPM scaling scan, while the red and green boxplots indicate the results for the Zimbabwean MSPM scan with a focus on planting and shelling respectively, the crosses mark the project leaders' answers. (For interpretation of the references to colour in this figure legend, the reader is referred to the web version of this article.)

Sample group indicators scores on the Scaling Ingredients are shown per country in Fig. 4, including the respective projects leaders' answers. The answers of individual participants within and between sample groups were variable, although within a country in most cases all sample groups answers hover around the same ratings. This is partly anticipated as the sample groups consist of people with previous experience on the matter and all have been exposed to the same priming presentation on scaling at the start of the workshop. Interestingly, even so, exceptions to this are the perception of Knowledge & Skills and to a lesser extent Evidence & Learning in Bangladesh, which the government participants rate visibly lower than the other groups, while in Mexico government actors deem Finance and Public sector governance better aligned for scaling MSPM comparing with private sector and project collaborators. In Zimbabwe, no hard discrepancies between sample groups answers exist, besides a lower rating from the private sector for Awareness in the MSPM Shelling and similar for Evidence & Learning in the case of the MSPM Planting.

### 3.2 Scalability assessment

#### 3.2.1 Technology/practice

The “Technology/Practice” ingredient refers to the different Mechanization Service Provider Models as promoted in the three case studies. The MSPM is an organizational innovation to make mechanization service for land preparation, planting, cultivation, and marketing accessible and affordable to smallholder farmers. In *MasAgro*, this is done through formal hiring centers - the MHCs - while individual ownership of machines and direct contracting with smallholder client farmers are promoted in CSISA-MI and FACASI. The attributes of the MSPM were found to be conducive for scaling especially because they have significant comparative advantages to alternative solutions in all three regions. The alternative service provider models include lending systems for animal draught power, or group ownership models that are generally very informally organized. Fleet service with four-wheeled tractors, typically through governmental programs, usually only provide land preparation services on large pieces of land. The latter requires land consolidation and established farmer organizations, which contrasts with the reality that 80% of rural farmers are smallholders and few are organized in groups. The MSPM aims to promote ownership and/or accountability of machinery appropriate to smallholders and facilitates sustainable and profitable business cases. Participants did indicate that the adoption of a MSP business is not easy as tactical question 1.3 scored rather low, because adopting the MSPM requires technical as well as business skills.

#### 3.2.2 Awareness and demand

Scoring 4.0 on average, issues around Awareness & Demand were found among the least inhibiting for scaling. Nevertheless, question 2.2 on access to information and effective communication channels (score 3.5) provoked a discussion that the organization of road shows or field demonstrations, and the development of advertising schemes and farmer exchange platforms to create awareness and demand are still largely reliant on the projects. Bangladesh may be a slight exception, as the country has a rich history of small-farm machinery use coupled with a high population density, high level of crop intensification, and relatively high demand for mechanized land preparation and irrigation (Mottaleb et al., 2016). CSISA-MI deliberately focuses on merging the MSPs with existing machinery platforms because farmers are largely familiar with service provider arrangements for primary tillage and irrigation (McHugh et al., 2019).

**Fig. 5:**
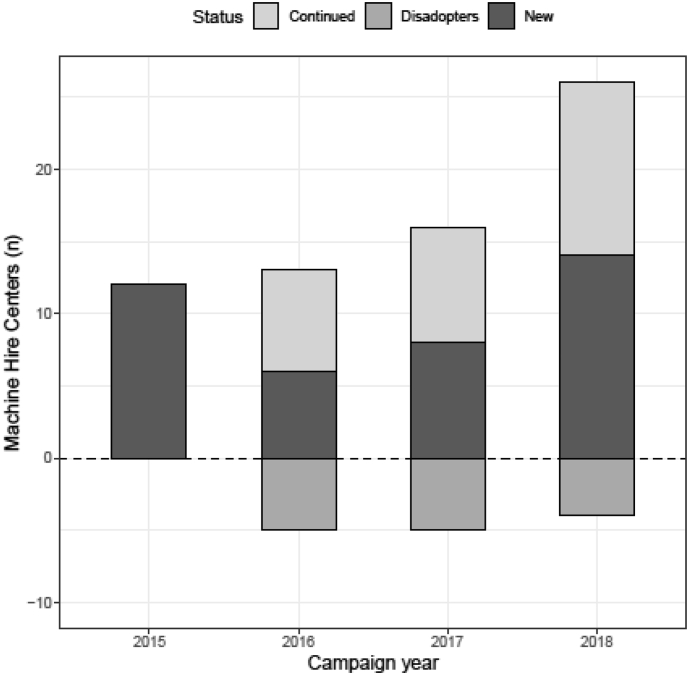
Machine Hire Centers in Mexico with annual status of newly established, continued centers, and dis-adopters.

An indicator for increasing demand is the growth in the number of MSPs, and with a high average score of 4.7 on question 2.3 it seems like this growing demand is noticeable in Mexico. *MasAgro* made the conditions for entering the MSPM very attractive (zero entry cost on machines) to trigger demand and awareness. On the other hand, this may explain that approximately one-third of service providers per year chose to not continue their MHC business pursuit (Fig. 5), while in Zimbabwe or in Bangladesh dis-adoption is not recorded. Reasons to dis-adopt are diverse, ranging from time limitations, to lack of service provider business skills and inability to maintain initial client base. Corresponding with the grand majority of projects that are promoting an innovation, dis-adoption is seldom studied (Chinseu et al., 2018) and a study on the specific reasons for dis-adoption in the three case-studies is yet to be commissioned.

#### 3.2.3 Business cases

The three cases promote the MSPM in order to stimulate rural entrepreneurship for machinery value chain actors, such as the dealers, service providers, and farmers. Attractive financial and economic propositions (i.e., the “business case”) for all these different actors is a prerequisite for successful scaling. Ample evidence is generated in the projects that show business cases for farmers that can reduce drudgery and production costs with multiple purpose machines (Baudron et al., 2015; Kahan et al., 2017). The business case for an MSP appears to depend partially on the client base to supply services to being well distributed over the year and within limited distance to reduce transaction costs.

The shelling MSPM in Zimbabwe scores highest for this ingredient with an average score of 4.1, confirming the business potential of post-harvest service provision in the area and again indicating that in very specific cases the technology need outweighs the provision mechanism. This might be the reason for the low score for Bangladesh’ case (3.4), where in the assessment no differentiation between offered services was made, since contrastingly client density is high in this region and new machinery options could piggyback on well-established service provider models for tillage and irrigation. Mexico's case results reach with a score of 3.8 a solid position, because acquisition and adaptation of machines is done by the *MasAgro* project. This however feeds the misconception of MSPs that business is only about finding customers and hence additional capital expenditures are not sufficiently incorporated.

Workshop participants indicated that machinery companies and dealerships are primarily only interested in introducing new equipment if they are assured of the potential to gain a new market segment. Looking back to Fig. 4, it is exactly here that the private sector participant opinions in Bangladesh differed the most on an individual level. In Mexico and Bangladesh, projects utilized geospatial information on cropping and wealth patterns to advise private sector partners where the strongest potential for sales and machinery adoption exist. Nevertheless, the tactical question 3.2 on availability of critical information to develop sound business cases scored lowest in those two countries.

#### 3.2.4 Value chain

The MSPs are heavily reliant on value chain actors for both equipment and skills. These are respectively the machinery value chain actors (machinery manufacturers, equipment and spare part – including maintenance – suppliers) and other actors that support their service provider business model, such as financial service providers, training providers, and business developers. In all case studies, CIMMYT is involved in influencing the service provider modalities and works towards integration within and among the two value chains. In Mexico, the MSPM requires that linked value chain actors are connected to the multi-stakeholder innovation hubs of the *MasAgro* framework. In the other cases, value chain integration relies on deliberate efforts of the projects to bring stakeholders together. There is little autonomous initiative to strengthen links among MSPs themselves, set standards, and uphold quality. With exception of the MSPM Shelling case in Zimbabwe, poor self-organization of the value chain actors scored lowest among all tactical questions for the Value Chain ingredient (question 4.4). In Mexico and Zimbabwe, value chains are affected by market distortions such as trade barriers (especially for spare parts) and restrictive intellectual property rights (IPR) for machine inventions. These constraints are particularly acute in Zimbabwe. In comparison, Bangladesh has a relatively flexible and supportive machinery import tariff policy (Mottaleb et al., 2016) and few formal IPR policies that conversely appear to encourage growth of machinery industries through competitive markets and “copycat” equipment, and this creates low barriers for entry into machinery dealing or service provider arrangements. The latter is strongly linked to high population density and thus potential farmer-clients. In general, demand and supply for appropriate mechanization services are high when population density is elevated, and small parcels, good access, and business support is provided. In Zimbabwe, demand and supply for mechanization services are comparatively low due to poor availability (poor local production), accessibility (low population density), and affordability (poor capacity to invest) of machinery. In Mexico, farmer demand for appropriate machinery options is relatively high, but supply is low because previous rural development efforts have historically focused on large-scale farms and machinery.

#### 3.2.5 Finance

Access to low-risk financing options appears to be an important bottleneck for smallholder farmers to purchase machinery and enter into service provider arrangements in all case studies (Fig. 3), with very low access to finance observed in Zimbabwe and in Mexico in particular. Agricultural banks in Zimbabwe, or loan facilities, for example, are hesitant to provide credit to smallholder farmers because of poor client information systems, lack of products adapted to agriculture, and the absence of land tenure titles for collateral. Poor confidence in the judiciary system to uphold contract rights is an additional concern. In Bangladesh -although efforts continue to focus on making financial service providers aware of two-wheeled tractor attachable machinery and how to support emerging service providers- access to finance is comparatively easy given the country's strong history of rural micro-lending services. Discussions with the sample groups in Bangladesh however indicate that financial service providers require education on agricultural machinery and mid-level lending programs that can support more expensive equipment like reapers or mini-combines. Only small and temporary subsidy programs are utilized to spur interest or high-profile sales of machinery in targeted geographies with weak markets. Conversely, long-standing governmental subsidy programs and low import tariffs for two-wheeled tractors and Chinese engines also reduce barriers to entry (Mottaleb et al., 2016). In Mexico, *MasAgro* covers all capital investment for machines in the hire centers, while the MHCs cover operational costs (use and maintenance), while on occasion in Zimbabwe through FACASI up to 50% of initial purchases was subsidized (this is for ‘pioneer service providers’ in particular areas where small mechanization is not yet known; elsewhere, service providers pay 100% of the value of the machines, though often over a period of three years). In both cases, financial support has at times resulted in unrealistic perceptions among potential MSPs of the start-up costs required to enter into business. In the case of Mexico, this can even result in general neglect of the provided machinery leading to cessation of the initial agreement followed by dis-adoption or contract retraction by CIMMYT. Although project technical support can help to reduce risks among early adopters, while benefiting first-mover companies, such programs can rarely be sustained beyond project funding cycles. When MSPs are engaged with farmer-clients in a more full-time professional capacity, as in Bangladesh, it appears more likely that commercial financial service providers will recognize the value of making loans for machinery and service provider businesses.

#### 3.2.6 Knowledge and skills

The MSP requires knowledge and skills in effective operation of farm machinery as well for managing a business. To address this, all case study projects deploy training materials and provide capacity strengthening in improved agronomy, operation, and maintenance of machines, as well as business skills for service providers. Although the public extension services have access to and use the training materials, the three projects use them in different ways. In Bangladesh, state extension officers are involved in the development and deployment of training curricula, and companies selling machines are targeted to provide after-sales services and informal trainings for their clients. Nevertheless, the government and project collaborators sample group in Bangladesh scored relatively low on this ingredient (2.4 and 2.9 respectively) compared to all other sample groups in Zimbabwe and Mexico (average score of 3.7 and above), indicative of their desire to further improve extension services related to farm machinery as elucidated by group discussion after the Scaling Scan. One of the conditions for support of *MasAgro* in Mexico is that each MHC also becomes a training center linked to the local innovation hub to promote appropriate mechanization services among farmers. In Zimbabwe, FACASI targets national vocational training centers to institutionalize and formalize trainings so that interested service providers, mechanics, and artisans can enroll independent of the project. Nonetheless, the costs for one five-day training is approximate USD 125 per person, which is not affordable to the entire target group, especially because at least two trainings per year are required. All three projects provide trainings for networks of mechanics and local artisans to execute repairs and make local adaptations to machines. Successful service providers require intensive one-on-one coaching, which is currently largely provided through each case study project, indicative of an important constraint that needs to be overcome to enable sustained scaling.

#### 3.2.7 Collaboration

In each of the case studies, both CIMMYT and core consortium partners (Government of Mexico and Zimbabwe, and iDE in Bangladesh) actively broker and facilitate collaboration among different actors (extension services, research partners, agricultural input dealers, other development programs) that support an environment for scaling. Additionally, CIMMYT plays a key role in linking governmental projects and NGOs that also aim to strengthen local capacity to access and use farm machinery. These collaborative efforts appear to be higher in Zimbabwe than in Mexico and Bangladesh, although our data indicate substantial room for improvement in all case studies (Fig. 3). Based on CIMMYT's operational history in each country, ties to machinery manufacturers, development organizations, and extension services tend to be strong, but collaborations with active participation of financial or marketing service providers are comparatively weak, yet crucial. Regular fora for structured collaboration among these actors was found only through the innovation hub platform of the *MasAgro* initiative in Mexico. CIMMYT also organizes occasional roundtables in Zimbabwe, and to a lesser extent in Bangladesh. In none of the case study countries, MSPs have created mechanisms to raise awareness or have organized associations to learn from each other. Especially in Bangladesh, the sample groups regard this as a major bottleneck for scaling (Fig. 4).

#### 3.2.8 Evidence and learning

**Fig. 6:**
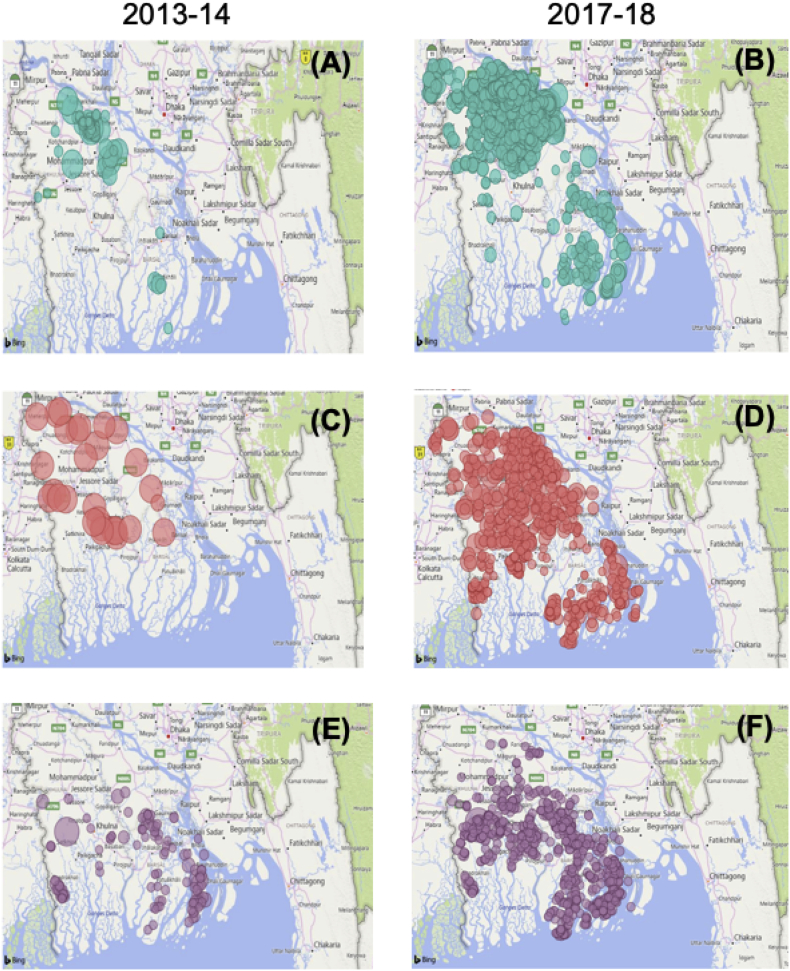
Example of the monitoring in growth of the number of two-wheeled tractor attachable seed and fertilizer drills (A and B), multi-crop reaper-harvesters (C and D), and axial flow irrigation pumps (E and F) used by mechanization service providers in 2013–14 compared to 2017–18 in southern Bangladesh (Map courtesy of Syed-Ur Rahman).

Each of the case studies scored three or above for the Evidence & Learning ingredient (Fig. 3) that measures how data and information are deployed to facilitate scaling processes. In Mexico and Bangladesh, mechanization projects use spatial databases to match potential users of machinery to farmers and cropping systems (Fig. 6). These approaches are also used to segment the client base for MSPs and incentivize them to match farmers' demand. In Mexico in particular, the geographical registration of machine usage is part of the commitment within the MHC agreement and could explain the elevated scoring on this ingredient (third most conducive among the scaling ingredients for the particular case study).

Despite the lower perception across sample groups, in Bangladesh, sales of machines to service providers without project involvement (e.g., facilitated third-party private sector partners) are also monitored. As machinery markets matured, an increasing number of companies began sales without having been approached by project partners. CSISA-MI has been successful in leveraging close to USD six million of private sector investment in appropriate machinery, with over 308,000 farmers purchasing machine services from just over 3400 service providers (McHugh et al., 2019).

**Fig. 7:**
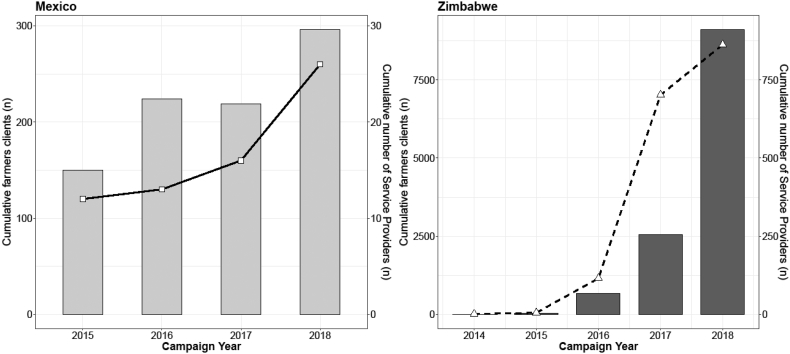
Evolution of established service providers (lines) and direct farmer beneficiaries (bars) from the mechanization service provider models in Mexico (left) and Zimbabwe (right).

Each of the three case study projects monitor service providers, including use of machines, number of hectares under machinery practices, and effects on the agronomic performance, as a means to measure progress of implementation or assess the orientation and trajectory of the proposed intervention for farmers and service providers. In Mexico and Zimbabwe this is done with a focus on the direct project beneficiaries (Fig. 7). In each case study, annual work plans are adjusted according to the outcomes of the monitoring data. Monitoring data, as shown in Fig. 2 and Fig. 7, revealed that the MSPMs promoted in different regions all have an exponential impact on the number of farmers or area upon which mechanization services became available as the projects advance, suggesting an apparent success of the chosen MSPM in each region.

#### 3.2.9 Leadership and management

Scaling of MSPMs benefits from leaders and spokespersons who position machinery in a broader context –for example, as part of climate smart agriculture, conservation agriculture, and sustainable intensification. Our case study projects provide leadership to strengthen service provider arrangements. This involves advocacy for machinery and service providers, engineering efforts to improve machinery performance, demand creation, and business intelligence supply as key incentives for private-sector-led sales and capacity development. While governments host and support the projects, they do not lead the scaling efforts. In Mexico, the promotion of MHCs benefits from strong political support to the overarching *MasAgro* program that promotes a range of sustainable intensification practices (IICA, 2016). In FACASI, mechanization service provider arrangements have been “picked up” and implemented by other agencies, including the United Nations Development Programme in eastern Zimbabwe, the *Deutsche Gesellschaft für Internationale Zusammenarbeit GmbH* (GIZ) in Ethiopia, the Development Fund of Norway in Malawi, and the International Fund for Agricultural Development in Rwanda and Zambia (Baudron, Personal Communication 2018, unpublished data).

In all regions, however, as confirmed by to lowest scoring question 9.2 on influential stakeholders (average score of 3.4), neither individual value chain actors, nor service providers or farmers' associations are sufficiently organized or incentivized to provide leadership in the coordination of scaling of MSPMs, and this suggest the need for a continued effort to make appropriate mechanization more appealing.

#### 3.2.10 Public sector governance

All governments in the case studies recognize that mechanization is crucial for rural development (Negrete, 2011; African Union Commision, 2014). All the case study projects interact with their respective agricultural ministries, and each country has relatively well-designed mechanization policies or strategies. Implementation is often difficult because of issues related to high taxes on metal and spare parts, interest rates, and transport regulations that lie with other ministries as economy, energy, labor, and livestock policies all influence smallholder farmers' access to machinery. Over *MasAgro*'s lifespan the government changed twice, which had significant impact on continuity, requiring project staff time investment to renew interest and shift priorities, and this is reflected in Fig. 3 where it accounts for the second least conducive scaling ingredient for the country. Subsidies are practically absent in Zimbabwe but comparatively strong and supported by favorable machinery import arrangements in Bangladesh. In general, participants were most critical about the government financing mechanisms not benefiting the scaling of MSPM with an average low score of 2.9 on question 10.4.

## 4 Conclusions

The three case study projects have the ambition to catalyze a service economy around agricultural mechanization driven by private sector actors. Each project developed a model that aims to be suitable to the operating context and with a potential to grow beyond the boundaries of the project. The *MasAgro* case study focuses on hiring services from organized project collaborators where the project provides the initial startup capital investment for machinery equipment. FACASI and CSISA-MI approach the service providers as independent entrepreneurs who benefit from technical and marketing support.

Within a matter of hours, the Scaling Scan tool helped three sample groups (government, private sector and project collaborators) have a structured exchange on what local ownership, leadership, sustainability and systems change means in their context. The results of the rapid evaluation, or scan, give strong indications for adaptations in project implementation strategies for MSPM to be viable at scale beyond the projects. However, they are a snapshot of opinions and interpretations that would be more robust when repeated over time and with bigger groups. More in-depth analysis is required to confirm these findings are indeed “the” critical issues and how they can be tackled before implementation strategies and project resources are adjusted. Although the Scaling Scan provides links to tools in its Annex that go more in-depth into specific ingredients and strategies, the scope of the tool is limited to a rapid scan.

The way the MSPMs are set up in each country are regarded relevant and much better than alternatives, such as lending or group ownership, and thus not likely to inhibit scaling. The major bottlenecks for scaling lie in the non-technological factors that constitute the enabling environment for the MSPM to thrive. Each of the projects invested considerably in capacity development on the use of machinery, but also on how to run a business. For example, the business cases for the MSPs and machinery dealers are strengthened in Mexico and Bangladesh because the projects provide targeting information on client segmentation and appropriate cropping systems for mechanization through geospatial and market data. In Zimbabwe, training programs are being adopted by vocational training centers and mechanization is now part of the university curriculum. Other donors in East Africa are promoting MSPMs and the private sector in Bangladesh invested almost USD six million in appropriate machinery. The State of Guanajuato in Mexico has made a commitment to pursue a similar strategy with a clear desire to stimulate the MSPM concept. However, results show that stakeholders close to the projects find that there are still considerable gaps to be filled in order for MSPMs to scale. The MSPs and associated value chain actors are still dependent on the projects to tackle major bottlenecks for scaling. Few solutions have been found to transition from project to market finance to sustainably facilitate machinery purchases and technical or business skills capacity development of service providers. Rather than supporting equipment purchases, smartly incentivizing potential clients to access mechanization services, while linking MSPs with machinery dealers and mechanics, might produce more satisfying results. In all three regions, supply inadequately keeps up with demand for appropriate mechanization services. More capacity development along the value chain is required to provide consistently high-quality services. The projects organize platforms for exchange, learning and awareness creation and governments may host platforms but they do not yet actively lead them, nor do associations of MSPs exist that could take that up.

Whereas typical monitoring, evaluation, and learning data tends to focus on quantification of adopters within the project context, the scaling assessment allowed a critical reflection on the dependencies the project has created in terms of financing, ownership and leadership of the scaling process. This raises important questions on what lies within the sphere of control of the project team, where new collaborations can fill gaps and what ultimately cannot be influenced by the projects. The study also shows how project interventions in one area can hamper development in other areas as illustrated by the *MasAgro* case where startup finance was provided to create demand for machines but thereby hampering initiatives around the business case and value chain ingredients. This shows that ingredients cannot be regarded in isolation of each other, they all give flavor to the dish. Furthermore, this study shows that the context in which an innovation is expected to scale is fundamental; referring to both the country context as well as the context created within the project through its design. It is important to note that the case studies were designed at least five years ago when scaling was associated with “technology adaptations to make it fit”, rather than with systems change and sustainability as it is today. Evidence for sustainable systems change can only be found beyond the project boundaries which calls for repeated assessments over time. The case projects are in their closing phases and findings are hence relevant for follow-up and other similar projects. We believe that many interventions can benefit from a more critical and participatory perspective on what it really takes to support an innovation to scale to a level where it contributes to positive rural transformation.

## Appendix A Supplementary data

Supplementary material 1 Image 1

Supplementary material 2 Image 2

Supplementary material 3 Image 3
