# Supplementary material for: Scaling agricultural mechanization services in smallholder farming systems: Case studies from sub-Saharan Africa, South Asia, and Latin America
Source: Agric Syst. 2020 Apr;180:102792. doi: 10.1016/j.agsy.2020.102792 (PMC7063696; doi:10.1016/j.agsy.2020.102792)
Supplement: Supplementary file 1 — Supplementary material 1 [file mmc1.docx]

**Supplementary Material 1**

**The Scaling Scan: Scaling ingredients and questions**

1. **Technology/Practice**
   1. Is your innovation relevant to your target group?
   2. Does the innovation have a comparative advantage over existing alternatives?
   3. Is the innovation easy to adopt?
   4. Is the innovation compatible with local circumstances and preferences?
2. **Awareness and Demand**
   1. Do important stakeholders recognize that a new technology/practice is necessary and desirable?
   2. Does the target group have access to information about the innovation and are there effective communication channels?
   3. Do you have evidence that demand for innovation is real and growing as anticipated?
   4. Can you distinguish segments of the target group for effective marketing of the innovation?
3. **Business Case**
   1. Are there viable business cases for the technology/practice for all actors along the value chain?
   2. Is enough information available to continue developing and sharpening business cases for the technology/practice?
   3. Do all value chain actors have a genuine interest to continue and improve the supply and use of the technology/practice?
   4. Is the business climate conducive to the business cases of all actors?
4. **Value Chain**
   1. Can the value chain provide/enable the technology/practice with the right quality, in the right quantity, and in a timely manner?
   2. Are relations between the various actors in the chain adequately developed?
   3. Is the overall performance of the value chain conducive to scaling?
   4. Are the target group and other value chain actors adequately organized?
5. **Finance**
   1. Can the target group finance the investment in, and operation of, the innovation?
   2. Are relevant financial mechanisms available, accessible, and affordable for all value chain actors?
   3. Is there sufficient and sustainable funding secured so that the scaling ambition can be achieved?
   4. Are financial risks acceptable for value chain actors and financial institutions/investors?
6. **Knowledge and Skills**
   1. Does the target group have the necessary knowledge and skills to use the innovation in the intended way?
   2. Are appropriate training materials and methods available to allow the target group and other value chain actors to adopt and promote the innovation?
   3. Are the right actors engaged to provide and improve the training programs necessary for sustainable adoption of the innovation?
   4. Is there an institutional environment in which actors (such as knowledge institutes) develop and improve the technology/practice within the national and local system?
7. **Collaboration**
   1. Are all actors relevant to scaling the innovation engaged?
   2. Are roles and responsibilities of key actors clear, accepted, and complementary?
   3. Are there effective networks or (sector) platforms for joint strategic direction-setting, advocacy, and creating buy-in?
   4. Do you have effective links with parallel initiatives or policy processes that could serve to scale the innovation?
8. **Evidence and Learning**
   1. Is there useful and credible data available on the impact and other parameters, which could help in understanding the scaling process?
   2. Is effective use being made of modern data and IT tools to support, analyze, share, and promote the innovation and to drive the change process?
   3. Are data and monitoring (including bottom-up/field data) effectively being used to steer the scaling process and change course where needed?
   4. Are you enabling institutional learning so the scaling process becomes more sustainable?
9. **Leadership and Management**
   1. Is the day-to-day leadership of the scaling process adequately established, recognized, and connected to the relevant actors?
   2. Are different actors and stakeholders sufficiently affecting the larger process and decision making?
   3. Are there adequate, influential, and compelling spokespersons, messengers, conveners, and power brokers for the innovation?
   4. Does the leadership support internal and external change management processes to achieve the organizational/institutional changes required?
10. **Public Sector Governance**
    1. Is the role of government in reaching your scaling ambition clearly defined?
    2. Are local and national strategies, policies, and regulations conducive to scaling the technology/practice?
    3. Are government agencies actively supporting scaling the innovation?
    4. Are relevant government-financing mechanisms (such as subsidies or tariffs) smart and can they be applied to benefit the scaling of the innovation?
