# Supplementary material for: Scaling agricultural mechanization services in smallholder farming systems: Case studies from sub-Saharan Africa, South Asia, and Latin America
Source: Agric Syst. 2020 Apr;180:102792. doi: 10.1016/j.agsy.2020.102792 (PMC7063696; doi:10.1016/j.agsy.2020.102792)
Supplement: Supplementary file 2 — Supplementary material 2 [file mmc2.docx]

**Supplementary Material 2**

**Statistical analysis on Scaling Ingredients indicator scores**

1. **Linear Mixed Model**

The scoring per ingredient in the Scaling Scan is analyzed by taking the average of the four corresponding tactical questions, building an **indicator score** for that ingredient. For clarity of representation, the Zimbabwean planting oriented MSPM and shelling MSPM are treated in separate MSPM scans, giving a total of 4 MSPMs that are considered in the scaling scan analysis. In order to detect **significant differences in responses from the participants across regions and sample groups**, a linear mixed model was constructed. Since the average scoring of the four corresponding tactical questions was used to create the indicator score, a normal distribution in the residuals per ingredient can be assumed and was confirmed by exploring histograms and QQ-plot of residuals (Figures A - C). The resulting linear mixed model *I* accounting for the country effect, sample group effect and interaction effect between both, is given as follows:

$$I : y_{ijk}=\mu+ \alpha_{i}+\beta_{j}+{\alpha\beta}_{ij}+ \varepsilon_{ijk}$$

with $y_{ijk}$ representing the indicator score for a specific scaling ingredient for MSPM *I*, sample group *j* and individual *k*; µ is the overall mean, $\alpha_{i}$ is the effect of the *i*-th MSPM (*i*=1, 2, 3, 4) and $\beta_{j}$ is the *j*-th sample group effect (j=1, 2, 3) , and ${\alpha\beta}_{ij}$ is the interaction effect between the *i*-the and *j*-th level of MSPM and sample group respectively, and finally $\varepsilon_{i}$ the error variance assumed as homogenous as they provided the best overall fit (AIC and BIC criterion^[[1]](#footnote-1)^ – Table 1).. An ANOVA was performed on the workshop results using this linear mixed model, and afterwards a t-test was run to separate means using SAS software (SAS, 2016).

All analyses were done with SAS/STAT 9.4 software using PROC MIXED and PROC GLM capabilities

(SAS Institute Inc. 2016. SAS/STAT® 14.3 User’s Guide. Cary, NC: SAS Institute Inc).

**Table 1.** Test of homogeneity of variance with linear mixed model I – homogeneity of score use by participant per MSPM

| Scaling Ingredients | Variance Type | -2 Res Log Likelihood | AIC (Smaller is Better) | AICC (Smaller is Better) | BIC (Smaller is Better) |
| --- | --- | --- | --- | --- | --- |
| Technology | **Homogeneity of variance** | 61.4 | **63.4** | **63.5** | **65.1** |
|  | Heterogeneity of variance | 55.6 | 63.6 | 64.8 | 71.4 |
| Awareness | Homogeneity of variance | 86.3 | 88.3 | 88.4 | **90.0** |
|  | *Heterogeneity of variance* | 78.3 | *86.3* | *87.5* | 94.1 |
| Business cases | **Homogeneity of variance** | 92.1 | **94.1** | **94.2** | **95.8** |
|  | Heterogeneity of variance | 89.7 | 97.7 | 98.8 | 105.5 |
| Value chain | **Homogeneity of variance** | 95.1 | **97.1** | **97.2** | **98.8** |
|  | Heterogeneity of variance | 89.1 | 97.1 | 98.2 | 104.9 |
| Finance | **Homogeneity of variance** | 101.2 | **103.2** | **103.3** | **104.9** |
|  | Heterogeneity of variance | 97.7 | 105.7 | 106.8 | 113.5 |
| Knowledge | **Homogeneity of variance** | 83.7 | **85.7** | **85.8** | **87.4** |
|  | Heterogeneity of variance | 82.1 | 90.1 | 91.3 | 97.9 |
| Collaboration | Homogeneity of variance | 92.5 | 94.5 | 94.6 | **96.2** |
|  | *Heterogeneity of variance* | 84.9 | *92.9* | *94.0* | 100.7 |
| Evidence | **Homogeneity of variance** | 98.6 | **100.6** | 100.7 | **102.3** |
|  | Heterogeneity of variance | 97.2 | 105.2 | *106.3* | 113.0 |
| Leadership | **Homogeneity of variance** | 97.1 | **99.1** | **99.3** | **100.8** |
|  | Heterogeneity of variance | 94.7 | 102.7 | 103.8 | 110.5 |
| Governance | **Homogeneity of variance** | 112.5 | **114.5** | **114.6** | **116.2** |
|  | Heterogeneity of variance | 107.3 | 115.3 | 116.5 | 123.1 |

1. **MSPM differences and MSPM by Sample group Comparison**

ANOVA results as presented in ***Table 2*** clearly showed, as expected, significant differences between countries on seven scaling ingredients. Only on Finance, Leadership and Governance differences are non-significant across countries. On scoring between sample groups no significant differences were found, and only for the indicator score of the Knowledge scaling ingredient a significant interaction was found between MSPM and Sample group.

**Table 2.** ANOVA results

| **Dependent** | **Source** | **DF** | **Sum of Squares** | **Mean Square** | **F Value** | **Pr > F** |
| --- | --- | --- | --- | --- | --- | --- |
| Technology | Sample group | 2 | 0.190 | 0.095 | 0.54 | 0.5881 |
|  | MSPM | 3 | 3.179 | 1.060 | 6.00 | **0.0018** |
|  | Sample group * MSPM | 6 | 0.629 | 0.105 | 0.59 | 0.7332 |
|  | Model | 11 | 4.045 | 0.368 | 2.08 | 0.045 |
|  | Error | 40 | 7.062 | 0.177 | _ | _ |
|  | Corrected Total | 51 | 11.107 | _ | _ | _ |
|  |  |  |  |  |  |  |
| Awareness | Sample group | 2 | 1.101 | 0.550 | 1.67 | 0.2003 |
|  | MSPM | 3 | 3.186 | 1.062 | 3.23 | **0.0323** |
|  | Sample group * MSPM | 6 | 3.020 | 0.503 | 1.53 | 0.1930 |
|  | Model | 11 | 7.950 | 0.723 | 2.2 | 0.034 |
|  | Error | 40 | 13.148 | 0.329 | _ | _ |
|  | Corrected Total | 51 | 21.098 | _ | _ | _ |
|  |  |  |  |  |  |  |
| Business Cases | Sample group | 2 | 0.483 | 0.241 | 0.64 | 0.5351 |
|  | MSPM | 3 | 4.062 | 1.354 | 3.56 | **0.0224** |
|  | Sample group * MSPM | 6 | 0.656 | 0.109 | 0.29 | 0.9394 |
|  | Model | 11 | 4.993 | 0.454 | 1.2 | 0.321 |
|  | Error | 40 | 15.192 | 0.380 | _ | _ |
|  | Corrected Total | 51 | 20.185 | _ | _ | _ |
|  |  |  |  |  |  |  |
| Value Chain | Sample group | 2 | 0.119 | 0.060 | 0.15 | 0.8651 |
|  | MSPM | 3 | 3.598 | 1.199 | 2.93 | **0.0452** |
|  | Sample group * MSPM | 6 | 2.110 | 0.352 | 0.86 | 0.5333 |
|  | Model | 11 | 5.291 | 0.481 | 1.17 | 0.335 |
|  | Error | 40 | 16.380 | 0.409 | _ | _ |
|  | Corrected Total | 51 | 21.671 | _ | _ | _ |
|  |  |  |  |  |  |  |
| Finance | Sample group | 2 | 1.573 | 0.786 | 1.65 | 0.2051 |
|  | MSPM | 3 | 1.258 | 0.419 | 0.88 | 0.4600 |
|  | Sample group * MSPM | 6 | 4.966 | 0.828 | 1.74 | 0.1379 |
|  | Model | 11 | 6.627 | 0.602 | 1.26 | 0.281 |
|  | Error | 40 | 19.082 | 0.477 | _ | _ |
|  | Corrected Total | 51 | 25.708 | _ | _ | _ |
|  |  |  |  |  |  |  |
| Knowledge | Sample group | 2 | 0.322 | 0.161 | 0.52 | 0.5975 |
|  | MSPM | 3 | 13.875 | 4.625 | 15.00 | **<.0001** |
|  | Sample group * MSPM | 6 | 5.021 | 0.837 | 2.71 | **0.0263** |
|  | Model | 11 | 17.899 | 1.627 | 5.28 | <.0001 |
|  | Error | 40 | 12.331 | 0.308 | _ | _ |
|  | Corrected Total | 51 | 30.231 | _ | _ | _ |
|  |  |  |  |  |  |  |
| Collaboration | Sample group | 2 | 0.566 | 0.283 | 0.74 | 0.4846 |
|  | MSPM | 3 | 6.578 | 2.193 | 5.72 | **0.0024** |
|  | Sample group * MSPM | 6 | 2.403 | 0.401 | 1.04 | 0.4116 |
|  | Model | 11 | 8.647 | 0.786 | 2.05 | 0.049 |
|  | Error | 40 | 15.341 | 0.384 | _ | _ |
|  | Corrected Total | 51 | 23.988 | _ | _ | _ |
|  |  |  |  |  |  |  |
| Evidence | Sample group | 2 | 0.202 | 0.101 | 0.23 | 0.7988 |
|  | MSPM | 3 | 10.149 | 3.383 | 7.57 | **0.0004** |
|  | Sample group * MSPM | 6 | 4.116 | 0.686 | 1.54 | 0.1916 |
|  | Model | 11 | 13.402 | 1.218 | 2.73 | 0.01 |
|  | Error | 40 | 17.868 | 0.447 | _ | _ |
|  | Corrected Total | 51 | 31.270 | _ | _ | _ |
|  |  |  |  |  |  |  |
| Leadership | Sample group | 2 | 0.701 | 0.350 | 0.81 | 0.4508 |
|  | MSPM | 3 | 2.851 | 0.950 | 2.20 | 0.1025 |
|  | Sample group * MSPM | 6 | 4.208 | 0.701 | 1.63 | 0.1649 |
|  | Model | 11 | 7.791 | 0.708 | 1.64 | 0.124 |
|  | Error | 40 | 17.241 | 0.431 | _ | _ |
|  | Corrected Total | 51 | 25.032 | _ | _ | _ |
|  |  |  |  |  |  |  |
| Governance | Sample group | 2 | 2.392 | 1.196 | 1.89 | 0.1642 |
|  | MSPM | 3 | 4.105 | 1.368 | 2.16 | 0.1073 |
|  | Sample group * MSPM | 6 | 3.446 | 0.574 | 0.91 | 0.4989 |
|  | Model | 11 | 9.207 | 0.837 | 1.32 | 0.248 |
|  | Error | 40 | 25.294 | 0.632 | _ | _ |
|  | Corrected Total | 51 | 34.501 | _ | _ | _ |

Subsequently, a T-test was used to compare MSPM and Sample group means. Results of this, for the six scaling ingredient that showed no MSPM by Sample group interaction are shown in ***Table 3***.

**Table 3.** T-test results indicating significances on scaling ingredient indicates scores between MSPMs

| **MSPM** | Technology | | Awareness | | Business Cases | | Value Chain | | Collaboration | | Evidence | |
| --- | --- | --- | --- | --- | --- | --- | --- | --- | --- | --- | --- | --- |
| Bangladesh | 4.18 | b | 4.05 | b | 3.39 | b | 3.07 | b | 3.01 | b | 3.02 | c |
| Mexico | 4.40 | b | 4.30 | b | 3.85 | ab | 3.67 | a | 3.40 | ab | 4.19 | a |
| Zimbabwe Planting | 4.04 | a | 3.59 | a | 3.46 | b | 3.51 | ab | 3.91 | a | 3.55 | bc |
| Zimbabwe Shelling | 4.73 | ab | 4.02 | ab | 4.10 | a | 3.75 | a | 3.82 | a | 3.93 | ab |

Finally, the results for the interaction effect on the Knowledge scaling ingredient is given below, indicating that the participant in Bangladesh (Bangladesh MSPM) scored the Knowledge ingredient significantly lower than the other countries’ participants (***Table 4***). Additionally, the government participants in Bangladesh scored significantly lower, compared to a intermediate perception of the project collaborators and the private sector group (***Table 5***).

**Table 4.** Results of Mechanization Service Provider Model by Sample group interaction effect sliced by MSPM for Knowledge

| **MSPM** | DF | Sum of Squares | Mean Square | F Value | Pr > F |
| --- | --- | --- | --- | --- | --- |
| Bangladesh | 2 | 2.408 | 1.204 | 3.91 | 0.0282 |
| Mexico | 2 | 0.699 | 0.349 | 1.13 | 0.3320 |
| Zimbabwe Planting | 2 | 1.467 | 0.734 | 2.38 | 0.1056 |
| Zimbabwe Shelling | 2 | 0.791 | 0.396 | 1.28 | 0.2883 |

**Table 5.** Results of Sample group by Mechanization Service Provider Model sliced by Sample group for knowledge

| **Sample Group** | DF | Sum of Squares | Mean Square | F Value | Pr > F |
| --- | --- | --- | --- | --- | --- |
| Government | 3 | 11.882 | 3.961 | 12.85 | **< .0001** |
| Private | 3 | 1.955 | 0.652 | 2.11 | 0.1137 |
| Project | 3 | 3.502 | 1.167 | 3.79 | **0.0176** |

1. **Additional figures**


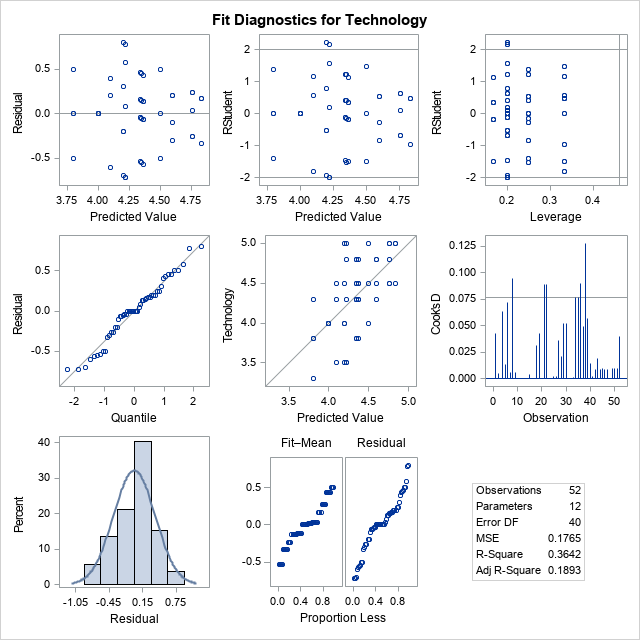

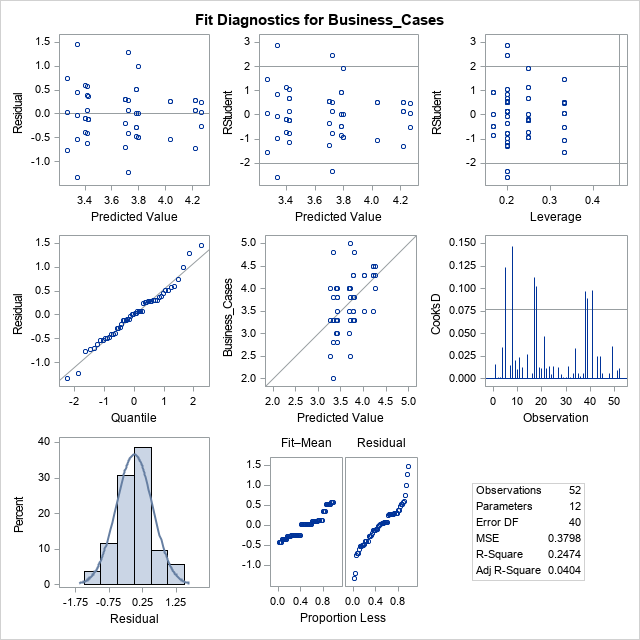

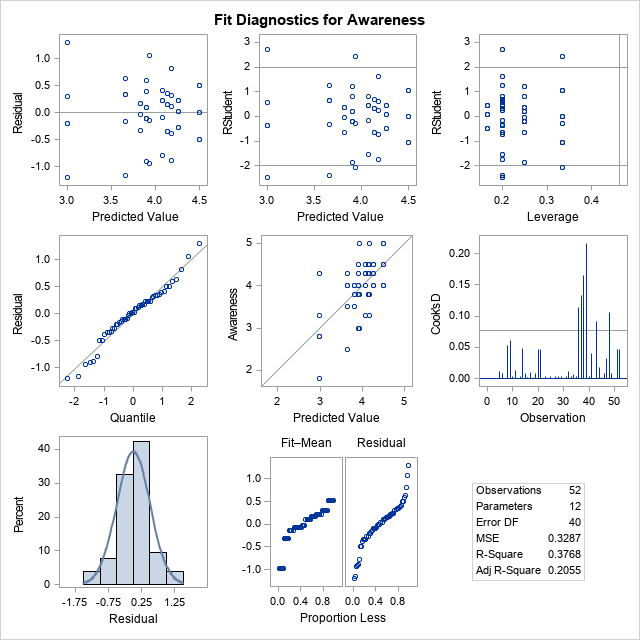

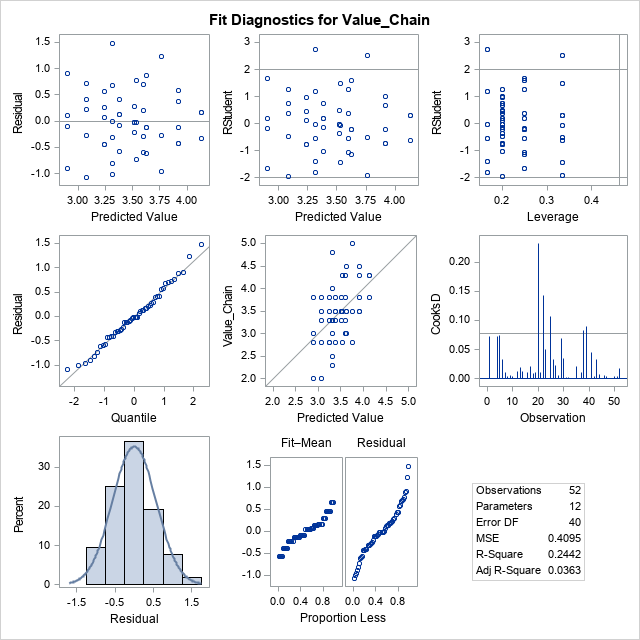


**Figure A.** Linear mixed model *I* fit diagnostics for indicator scoring per scaling ingredient (Technology/Practice, Awareness, Business Cases and Value Chain), indicating normal distribution of the residuals


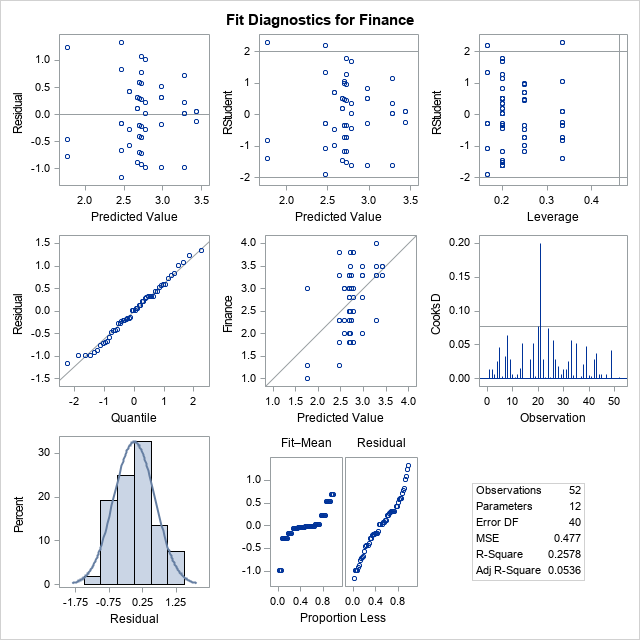

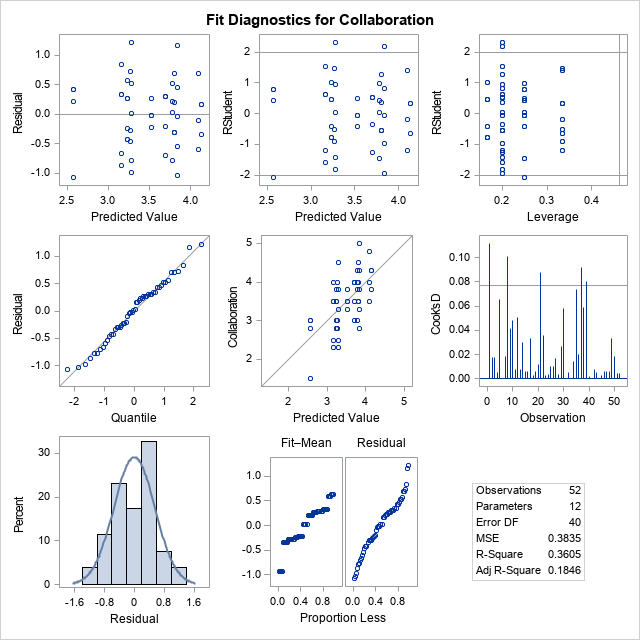

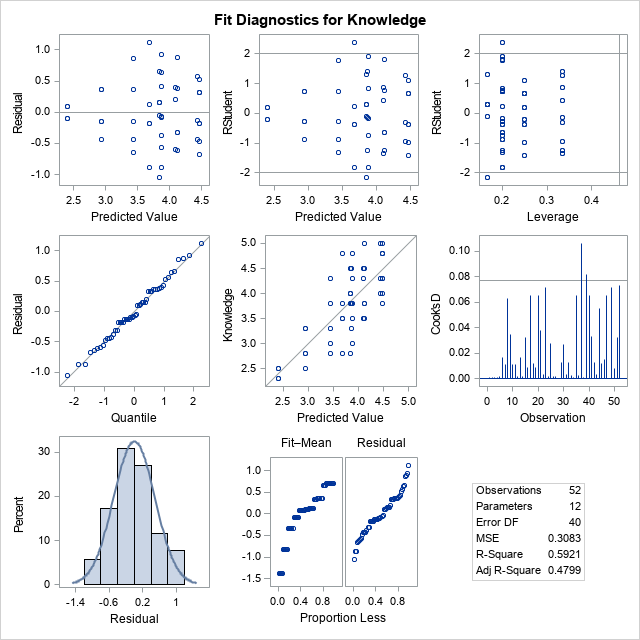

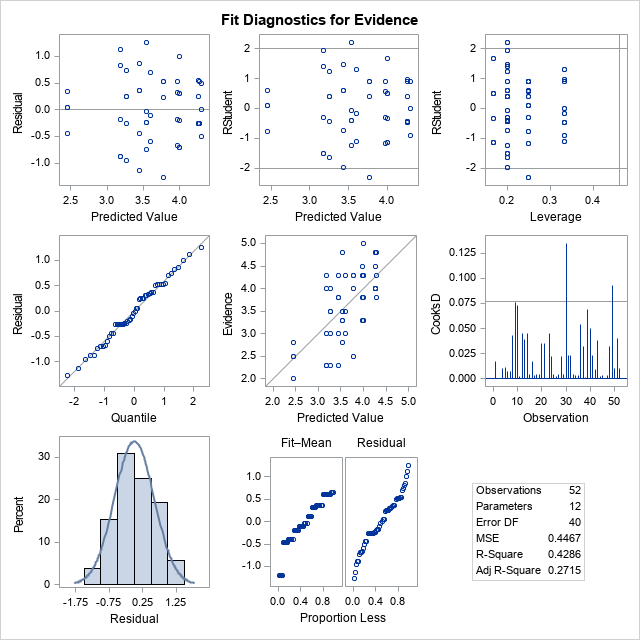


**Figure B.** Linear mixed model *I* fit diagnostics for indicator scoring per scaling ingredient (Finance, Knowledge, Collaboration and Evidence & Learning), indicating normal distribution of the residuals


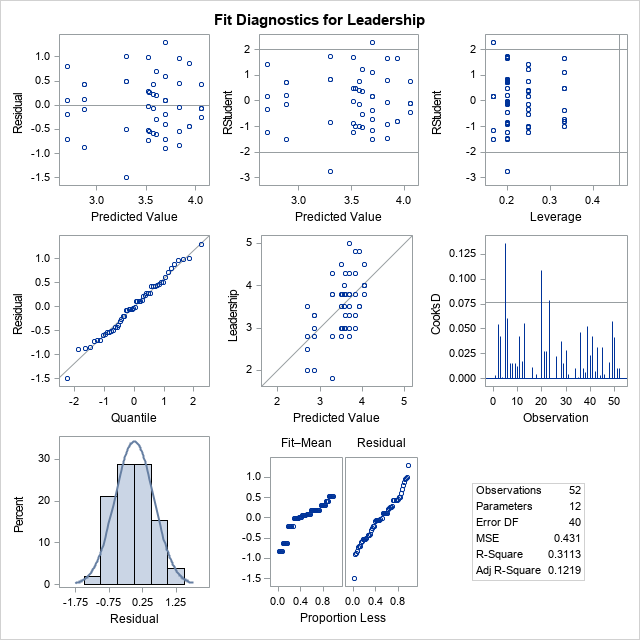

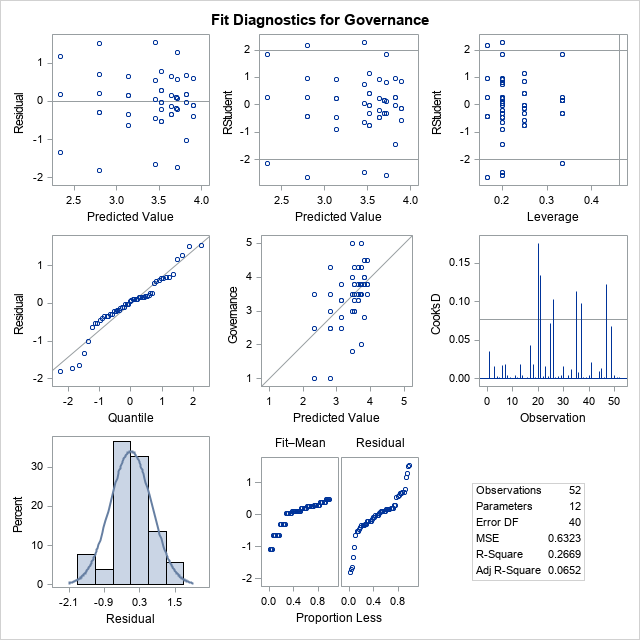


**Figure C.** Linear mixed model *I* fit diagnostics for indicator scoring per scaling ingredient (Leadership and Public Sector Governance), indicating normal distribution of the residuals

1. Eight out of ten scaling ingredient indicators showed homogeneity of variance, remaining two show no clear resolution, hence to harmonize analysis a homogenous error variance structure is used [↑](#footnote-ref-1)
