# Supplementary material for: Scaling agricultural mechanization services in smallholder farming systems: Case studies from sub-Saharan Africa, South Asia, and Latin America
Source: Agric Syst. 2020 Apr;180:102792. doi: 10.1016/j.agsy.2020.102792 (PMC7063696; doi:10.1016/j.agsy.2020.102792)
Supplement: Supplementary file 3 — Supplementary material 3 [file mmc3.docx]

**Supplementary Material 3**

**Scaling Workshop results: Scoring on Individual tactical questions**

Scaling workshops results, as scores given per tactical question are provided in this document. First heat maps present an overview of the overall ratings per country (B for Bangladesh, M for Mexico, Z for Zimbabwe) and per individual, with an indication of the sample group the participant belongs to (G for government, P for private sector and C for project collaborators). In Zimbabwe, scores for both scaling scans (MSPM Planting and MSPM Shelling) are given. At the end of the document, the full dataset of scores is provided.

1. **Bangladesh**


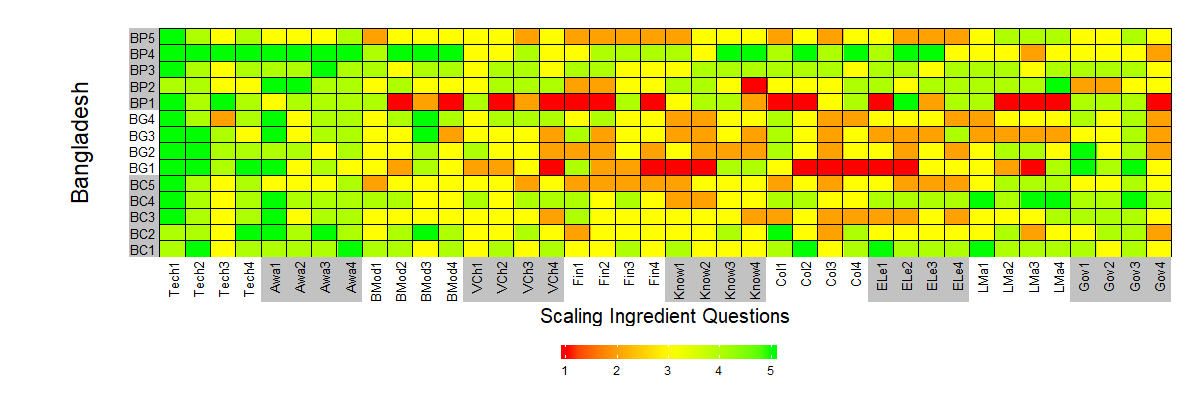


1. **Mexico**


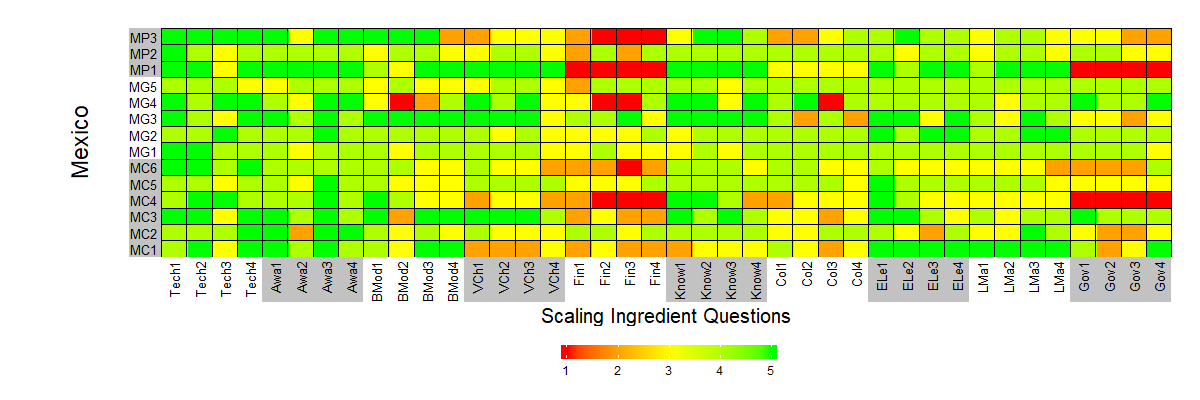


1. **Zimbabwe**


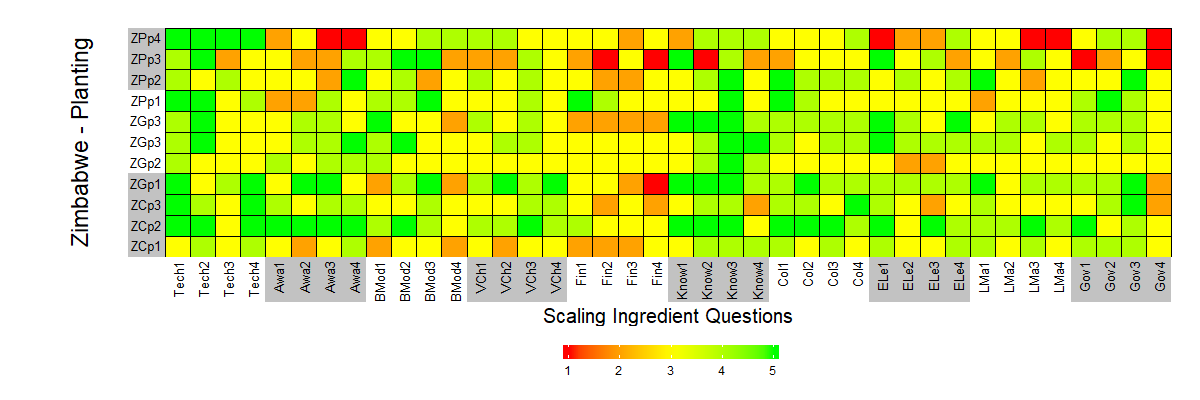

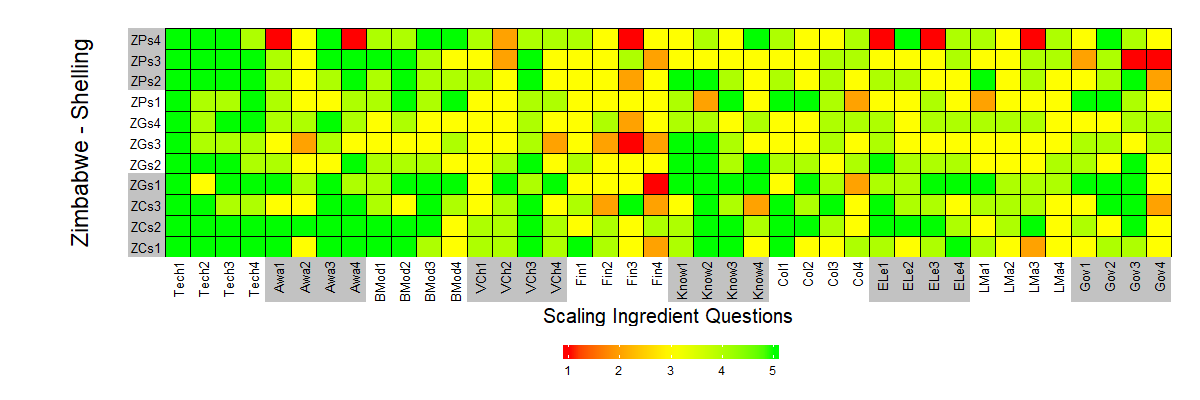


1. **Full dataset from 3 workshops with scoring on Scaling Ingredient tactical questions**

|  | Technology | | | | Awareness | | | | Business Cases | | | | Value Chain | | | | Finance | | | | Knowledge | | | | Collaboration | | | | Evidence | | | | Leadership | | | | Governance | | | |
| --- | --- | --- | --- | --- | --- | --- | --- | --- | --- | --- | --- | --- | --- | --- | --- | --- | --- | --- | --- | --- | --- | --- | --- | --- | --- | --- | --- | --- | --- | --- | --- | --- | --- | --- | --- | --- | --- | --- | --- | --- |
|  | 1.1 | 1.2 | 1.3 | 1.4 | 2.1 | 2.2 | 2.3 | 2.4 | 3.1 | 3.2 | 3.3 | 3.4 | 4.1 | 4.2 | 4.3 | 4.4 | 5.1 | 5.2 | 5.3 | 5.4 | 6.1 | 6.2 | 6.3 | 6.4 | 7.1 | 7.2 | 7.3 | 7.4 | 8.1 | 8.2 | 8.3 | 8.4 | 9.1 | 9.2 | 9.3 | 9.4 | 10.1 | 10.2 | 10.3 | 10.4 |
| Bangladesh | | | | |  |  |  |  |  |  |  |  |  |  |  |  |  |  |  |  |  |  |  |  |  |  |  |  |  |  |  |  |  |  |  |  |  |  |  |  |
| BG1 | 5 | 5 | 4 | 5 | 5 | 3 | 4 | 3 | 3 | 2 | 4 | 3 | 2 | 2 | 3 | 1 | 4 | 2 | 2 | 1 | 1 | 1 | 4 | 4 | 3 | 1 | 1 | 1 | 1 | 1 | 3 | 3 | 3 | 2 | 1 | 4 | 5 | 4 | 5 | 3 |
| BG2 | 5 | 5 | 4 | 4 | 4 | 4 | 3 | 4 | 3 | 3 | 4 | 3 | 4 | 3 | 3 | 2 | 2 | 2 | 3 | 2 | 3 | 2 | 2 | 2 | 4 | 3 | 2 | 3 | 3 | 2 | 3 | 2 | 3 | 4 | 4 | 3 | 5 | 3 | 4 | 2 |
| BG3 | 5 | 5 | 4 | 3 | 5 | 3 | 4 | 4 | 3 | 3 | 5 | 2 | 3 | 3 | 3 | 2 | 4 | 2 | 3 | 3 | 2 | 2 | 3 | 2 | 4 | 3 | 2 | 3 | 2 | 2 | 2 | 4 | 2 | 2 | 2 | 2 | 3 | 3 | 4 | 2 |
| BG4 | 5 | 4 | 2 | 4 | 5 | 3 | 4 | 4 | 3 | 4 | 5 | 4 | 3 | 4 | 4 | 4 | 3 | 4 | 3 | 3 | 2 | 2 | 3 | 3 | 4 | 3 | 2 | 2 | 3 | 3 | 3 | 2 | 2 | 3 | 3 | 3 | 4 | 3 | 4 | 2 |
| BP1 | 5 | 4 | 5 | 4 | 3 | 4 | 4 | 4 | 4 | 1 | 2 | 1 | 4 | 1 | 2 | 1 | 1 | 1 | 4 | 1 | 3 | 4 | 4 | 2 | 1 | 1 | 3 | 4 | 1 | 5 | 2 | 4 | 4 | 1 | 1 | 1 | 4 | 4 | 4 | 1 |
| BP2 | 4 | 4 | 3 | 3 | 5 | 5 | 4 | 4 | 3 | 4 | 3 | 3 | 3 | 4 | 4 | 4 | 2 | 2 | 3 | 3 | 4 | 4 | 3 | 1 | 3 | 3 | 3 | 4 | 4 | 3 | 4 | 4 | 4 | 4 | 4 | 5 | 2 | 2 | 3 | 3 |
| BP3 | 5 | 4 | 3 | 4 | 4 | 4 | 5 | 4 | 4 | 3 | 4 | 4 | 3 | 4 | 4 | 3 | 4 | 4 | 3 | 3 | 3 | 4 | 4 | 4 | 4 | 4 | 4 | 3 | 4 | 4 | 3 | 4 | 4 | 4 | 3 | 4 | 4 | 4 | 4 | 3 |
| BP4 | 5 | 5 | 5 | 5 | 5 | 5 | 5 | 5 | 4 | 5 | 5 | 5 | 3 | 3 | 4 | 3 | 3 | 4 | 4 | 4 | 4 | 3 | 5 | 5 | 4 | 5 | 4 | 5 | 4 | 5 | 5 | 3 | 3 | 3 | 2 | 3 | 3 | 3 | 3 | 2 |
| BP5 | 5 | 4 | 3 | 4 | 3 | 3 | 3 | 4 | 2 | 3 | 3 | 3 | 3 | 3 | 2 | 3 | 2 | 2 | 2 | 2 | 2 | 3 | 3 | 3 | 2 | 3 | 2 | 3 | 3 | 2 | 2 | 2 | 3 | 4 | 4 | 4 | 3 | 3 | 4 | 3 |
| BC1 | 4 | 5 | 3 | 4 | 4 | 4 | 4 | 5 | 4 | 4 | 3 | 4 | 3 | 4 | 3 | 4 | 3 | 3 | 4 | 3 | 4 | 3 | 3 | 3 | 4 | 5 | 3 | 4 | 5 | 4 | 4 | 4 | 5 | 4 | 4 | 4 | 4 | 3 | 4 | 3 |
| BC2 | 4 | 4 | 3 | 5 | 5 | 4 | 5 | 4 | 3 | 4 | 5 | 4 | 3 | 3 | 4 | 3 | 2 | 3 | 3 | 3 | 3 | 3 | 4 | 3 | 5 | 3 | 2 | 4 | 3 | 3 | 3 | 3 | 3 | 4 | 2 | 3 | 3 | 4 | 4 | 2 |
| BC3 | 5 | 4 | 3 | 4 | 5 | 3 | 4 | 4 | 3 | 3 | 3 | 3 | 3 | 3 | 3 | 2 | 4 | 3 | 3 | 3 | 3 | 3 | 3 | 2 | 2 | 3 | 2 | 2 | 2 | 2 | 3 | 2 | 3 | 3 | 3 | 4 | 4 | 4 | 4 | 3 |
| BC4 | 5 | 4 | 3 | 4 | 5 | 4 | 4 | 4 | 4 | 4 | 3 | 3 | 4 | 3 | 4 | 4 | 4 | 3 | 4 | 3 | 2 | 2 | 3 | 3 | 4 | 4 | 3 | 3 | 4 | 4 | 4 | 4 | 5 | 4 | 5 | 5 | 4 | 4 | 5 | 4 |
| BC5 | 5 | 4 | 3 | 4 | 3 | 3 | 3 | 4 | 2 | 3 | 3 | 3 | 3 | 3 | 2 | 3 | 2 | 2 | 2 | 2 | 2 | 3 | 3 | 3 | 2 | 3 | 2 | 3 | 3 | 2 | 2 | 2 | 3 | 4 | 4 | 4 | 3 | 3 | 4 | 3 |
| Mexico | | | | |  |  |  |  |  |  |  |  |  |  |  |  |  |  |  |  |  |  |  |  |  |  |  |  |  |  |  |  |  |  |  |  |  |  |  |  |
| MG1 | 5 | 5 | 4 | 4 | 4 | 3 | 4 | 4 | 4 | 3 | 4 | 4 | 4 | 4 | 4 | 3 | 3 | 4 | 3 | 3 | 3 | 4 | 3 | 4 | 4 | 4 | 4 | 4 | 4 | 4 | 4 | 4 | 4 | 4 | 4 | 4 | 4 | 4 | 4 | 3 |
| MG2 | 4 | 4 | 5 | 4 | 4 | 4 | 5 | 4 | 4 | 4 | 4 | 4 | 4 | 3 | 4 | 3 | 3 | 3 | 3 | 4 | 3 | 4 | 4 | 4 | 4 | 4 | 4 | 4 | 5 | 4 | 5 | 5 | 4 | 4 | 5 | 5 | 4 | 4 | 4 | 4 |
| MG3 | 5 | 4 | 3 | 5 | 5 | 4 | 5 | 4 | 5 | 5 | 5 | 5 | 5 | 5 | 5 | 3 | 4 | 4 | 5 | 3 | 5 | 5 | 5 | 5 | 4 | 2 | 4 | 2 | 5 | 5 | 3 | 5 | 4 | 3 | 5 | 4 | 3 | 3 | 2 | 3 |
| MG4 | 5 | 4 | 5 | 5 | 4 | 3 | 5 | 5 | 3 | 1 | 2 | 4 | 5 | 4 | 5 | 3 | 3 | 1 | 1 | 4 | 5 | 5 | 3 | 5 | 4 | 5 | 1 | 4 | 4 | 4 | 4 | 4 | 4 | 3 | 4 | 4 | 5 | 4 | 4 | 5 |
| MG5 | 4 | 4 | 4 | 3 | 3 | 4 | 4 | 4 | 3 | 4 | 3 | 3 | 3 | 4 | 4 | 3 | 2 | 4 | 4 | 4 | 4 | 4 | 3 | 4 | 4 | 4 | 4 | 4 | 4 | 4 | 4 | 4 | 4 | 4 | 4 | 4 | 4 | 4 | 4 | 4 |
| MP1 | 5 | 5 | 3 | 5 | 5 | 5 | 5 | 5 | 4 | 3 | 5 | 5 | 5 | 5 | 5 | 5 | 1 | 1 | 1 | 1 | 5 | 5 | 5 | 5 | 3 | 3 | 3 | 3 | 5 | 4 | 5 | 5 | 4 | 5 | 5 | 5 | 1 | 1 | 1 | 1 |
| MP2 | 5 | 4 | 3 | 4 | 4 | 4 | 4 | 4 | 3 | 4 | 4 | 3 | 3 | 4 | 4 | 3 | 2 | 4 | 2 | 4 | 4 | 4 | 4 | 4 | 4 | 4 | 4 | 4 | 4 | 3 | 4 | 4 | 3 | 4 | 4 | 3 | 4 | 4 | 3 | 3 |
| MP3 | 5 | 5 | 5 | 5 | 5 | 3 | 5 | 5 | 5 | 5 | 5 | 2 | 2 | 3 | 3 | 3 | 2 | 1 | 1 | 1 | 3 | 5 | 5 | 4 | 2 | 2 | 3 | 4 | 4 | 5 | 4 | 4 | 3 | 4 | 4 | 3 | 3 | 3 | 2 | 2 |
| MC1 | 4 | 5 | 3 | 5 | 5 | 4 | 5 | 4 | 4 | 3 | 5 | 5 | 2 | 2 | 2 | 3 | 2 | 3 | 2 | 2 | 2 | 3 | 3 | 3 | 4 | 3 | 2 | 3 | 5 | 5 | 5 | 5 | 5 | 5 | 5 | 5 | 4 | 2 | 3 | 5 |
| MC2 | 4 | 4 | 4 | 5 | 5 | 2 | 5 | 5 | 4 | 3 | 4 | 3 | 4 | 3 | 3 | 3 | 4 | 4 | 3 | 4 | 4 | 4 | 4 | 4 | 3 | 3 | 4 | 4 | 4 | 3 | 2 | 4 | 3 | 3 | 5 | 4 | 3 | 2 | 2 | 3 |
| MC3 | 5 | 5 | 3 | 5 | 5 | 4 | 5 | 4 | 5 | 2 | 5 | 5 | 5 | 5 | 5 | 4 | 2 | 3 | 2 | 2 | 5 | 4 | 5 | 4 | 3 | 3 | 2 | 3 | 5 | 5 | 4 | 3 | 4 | 3 | 4 | 4 | 5 | 4 | 4 | 4 |
| MC4 | 4 | 5 | 5 | 4 | 4 | 4 | 5 | 4 | 5 | 4 | 3 | 3 | 2 | 3 | 3 | 2 | 2 | 1 | 1 | 1 | 5 | 5 | 4 | 2 | 2 | 3 | 3 | 3 | 5 | 4 | 3 | 3 | 3 | 3 | 3 | 3 | 1 | 1 | 1 | 1 |
| MC5 | 4 | 4 | 3 | 4 | 4 | 3 | 5 | 4 | 4 | 3 | 3 | 3 | 4 | 4 | 4 | 4 | 3 | 3 | 3 | 4 | 4 | 4 | 4 | 4 | 4 | 4 | 4 | 3 | 5 | 4 | 4 | 4 | 4 | 4 | 4 | 3 | 3 | 3 | 3 | 3 |
| MC6 | 5 | 5 | 4 | 5 | 4 | 4 | 4 | 4 | 4 | 4 | 3 | 3 | 4 | 3 | 3 | 2 | 2 | 2 | 1 | 2 | 4 | 4 | 4 | 3 | 4 | 4 | 3 | 3 | 4 | 3 | 3 | 3 | 3 | 3 | 3 | 2 | 2 | 2 | 2 | 4 |
| Zimbabwe Planting | | | | |  |  |  |  |  |  |  |  |  |  |  |  |  |  |  |  |  |  |  |  |  |  |  |  |  |  |  |  |  |  |  |  |  |  |  |  |
| ZG1 | 5 | 3 | 4 | 5 | 3 | 5 | 5 | 3 | 2 | 4 | 5 | 2 | 4 | 5 | 4 | 5 | 3 | 3 | 2 | 1 | 5 | 5 | 5 | 4 | 4 | 5 | 4 | 4 | 4 | 4 | 4 | 4 | 5 | 3 | 4 | 4 | 4 | 4 | 5 | 2 |
| ZG2 | 4 | 3 | 3 | 3 | 4 | 3 | 4 | 4 | 4 | 3 | 3 | 3 | 3 | 3 | 3 | 3 | 3 | 3 | 3 | 3 | 3 | 4 | 5 | 4 | 3 | 3 | 3 | 3 | 3 | 2 | 2 | 3 | 3 | 3 | 3 | 3 | 3 | 3 | 3 | 3 |
| ZG3 | 4 | 5 | 3 | 3 | 3 | 4 | 4 | 5 | 4 | 5 | 3 | 3 | 3 | 4 | 4 | 3 | 3 | 3 | 3 | 3 | 3 | 4 | 5 | 5 | 4 | 3 | 4 | 4 | 5 | 4 | 4 | 4 | 4 | 4 | 3 | 4 | 4 | 3 | 4 | 3 |
| ZG4 | 4 | 5 | 3 | 3 | 3 | 4 | 4 | 3 | 5 | 3 | 3 | 2 | 4 | 3 | 4 | 3 | 2 | 2 | 2 | 2 | 5 | 5 | 5 | 4 | 4 | 4 | 4 | 4 | 5 | 4 | 3 | 5 | 3 | 4 | 4 | 3 | 4 | 4 | 4 | 3 |
| ZP1 | 5 | 5 | 3 | 4 | 2 | 2 | 4 | 3 | 4 | 4 | 5 | 3 | 3 | 3 | 4 | 3 | 5 | 4 | 3 | 3 | 3 | 3 | 5 | 3 | 5 | 4 | 3 | 3 | 3 | 3 | 3 | 3 | 2 | 3 | 3 | 3 | 4 | 5 | 4 | 3 |
| ZP2 | 4 | 3 | 4 | 3 | 3 | 3 | 2 | 5 | 3 | 4 | 2 | 3 | 4 | 3 | 4 | 3 | 3 | 2 | 2 | 3 | 3 | 4 | 5 | 3 | 5 | 4 | 4 | 4 | 3 | 4 | 3 | 4 | 5 | 3 | 2 | 3 | 3 | 3 | 5 | 3 |
| ZP3 | 4 | 5 | 2 | 3 | 3 | 2 | 2 | 4 | 4 | 5 | 5 | 2 | 2 | 2 | 4 | 3 | 2 | 1 | 3 | 1 | 5 | 0 | 4 | 2 | 2 | 3 | 3 | 3 | 5 | 3 | 4 | 2 | 3 | 2 | 4 | 3 | 1 | 2 | 3 | 1 |
| ZP4 | 5 | 5 | 5 | 5 | 2 | 3 | 1 | 1 | 3 | 3 | 4 | 4 | 4 | 4 | 3 | 3 | 3 | 3 | 2 | 3 | 2 | 4 | 4 | 4 | 3 | 3 | 3 | 4 | 1 | 2 | 2 | 4 | 3 | 3 | 1 | 1 | 3 | 4 | 4 | 1 |
| ZP5 | 4 | 5 | 5 | 5 | 5 | 4 | 3 | 5 | 5 | 3 | 4 | 4 | 4 | 4 | 4 | 3 | 3 | 3 | 3 | 3 | 4 | 5 | 5 | 5 | 5 | 5 | 5 | 5 | 4 | 4 | 4 | 4 | 4 | 3 | 3 | 3 | 5 | 5 | 5 | 5 |
| ZC1 | 3 | 4 | 3 | 4 | 3 | 2 | 3 | 4 | 2 | 3 | 3 | 2 | 3 | 2 | 3 | 3 | 2 | 2 | 2 | 3 | 3 | 4 | 4 | 4 | 4 | 3 | 4 | 3 | 4 | 3 | 3 | 4 | 4 | 3 | 4 | 3 | 4 | 4 | 4 | 3 |
| ZC2 | 5 | 5 | 3 | 5 | 5 | 5 | 5 | 5 | 4 | 5 | 4 | 3 | 4 | 4 | 5 | 4 | 4 | 3 | 3 | 3 | 5 | 5 | 5 | 3 | 5 | 5 | 5 | 4 | 5 | 3 | 5 | 4 | 4 | 4 | 5 | 4 | 5 | 3 | 4 | 3 |
| ZC3 | 5 | 4 | 3 | 5 | 4 | 3 | 4 | 4 | 3 | 3 | 4 | 3 | 3 | 3 | 4 | 4 | 3 | 2 | 3 | 2 | 3 | 4 | 4 | 2 | 4 | 4 | 3 | 5 | 4 | 3 | 2 | 3 | 4 | 3 | 3 | 3 | 3 | 4 | 5 | 2 |
| Zimbabwe Shelling | | | | |  |  |  |  |  |  |  |  |  |  |  |  |  |  |  |  |  |  |  |  |  |  |  |  |  |  |  |  |  |  |  |  |  |  |  |  |
| ZG1 | 5 | 3 | 5 | 5 | 5 | 4 | 5 | 4 | 4 | 5 | 5 | 5 | 3 | 5 | 4 | 5 | 3 | 3 | 3 | 1 | 5 | 5 | 5 | 5 | 3 | 5 | 4 | 2 | 4 | 4 | 5 | 5 | 5 | 4 | 4 | 4 | 5 | 5 | 5 | 3 |
| ZG2 | 5 | 5 | 5 | 4 | 4 | 3 | 3 | 5 | 4 | 4 | 4 | 3 | 3 | 4 | 5 | 3 | 4 | 3 | 3 | 3 | 5 | 5 | 4 | 5 | 4 | 4 | 3 | 4 | 5 | 4 | 4 | 4 | 3 | 3 | 3 | 4 | 4 | 3 | 5 | 3 |
| ZG3 | 5 | 4 | 4 | 4 | 3 | 2 | 4 | 3 | 3 | 3 | 3 | 4 | 3 | 3 | 4 | 2 | 3 | 2 | 1 | 2 | 5 | 5 | 4 | 3 | 3 | 3 | 4 | 3 | 4 | 3 | 3 | 3 | 3 | 3 | 3 | 3 | 4 | 4 | 3 | 4 |
| ZG4 | 5 | 4 | 5 | 5 | 4 | 4 | 5 | 4 | 3 | 4 | 3 | 3 | 3 | 3 | 4 | 3 | 3 | 4 | 2 | 3 | 4 | 4 | 3 | 4 | 3 | 3 | 4 | 4 | 4 | 3 | 4 | 4 | 4 | 4 | 4 | 3 | 3 | 3 | 4 | 4 |
| ZP1 | 5 | 4 | 4 | 5 | 4 | 3 | 3 | 4 | 4 | 5 | 4 | 5 | 3 | 3 | 4 | 4 | 3 | 3 | 3 | 3 | 4 | 2 | 5 | 3 | 5 | 5 | 4 | 2 | 3 | 3 | 4 | 3 | 2 | 3 | 3 | 3 | 5 | 5 | 4 | 3 |
| ZP2 | 5 | 5 | 5 | 5 | 4 | 3 | 4 | 5 | 4 | 5 | 4 | 4 | 4 | 3 | 5 | 3 | 3 | 3 | 2 | 3 | 5 | 5 | 4 | 3 | 4 | 3 | 4 | 3 | 4 | 4 | 3 | 3 | 5 | 3 | 4 | 3 | 3 | 4 | 5 | 2 |
| ZP3 | 5 | 5 | 5 | 5 | 4 | 3 | 5 | 5 | 5 | 5 | 4 | 3 | 3 | 2 | 5 | 3 | 3 | 3 | 4 | 2 | 3 | 3 | 3 | 3 | 3 | 3 | 4 | 4 | 3 | 3 | 3 | 4 | 3 | 3 | 4 | 4 | 2 | 4 | 1 | 1 |
| ZP4 | 5 | 5 | 5 | 4 | 1 | 3 | 5 | 1 | 4 | 4 | 5 | 5 | 4 | 2 | 4 | 4 | 4 | 3 | 1 | 3 | 3 | 4 | 3 | 5 | 4 | 3 | 3 | 4 | 1 | 5 | 1 | 4 | 4 | 3 | 1 | 4 | 3 | 5 | 4 | 3 |
| ZP5 | 4 | 5 | 5 | 4 | 5 | 3 | 3 | 5 | 4 | 4 | 4 | 2 | 3 | 3 | 4 | 4 | 4 | 1 | 1 | 1 | 4 | 5 | 5 | 5 | 5 | 5 | 4 | 4 | 5 | 5 | 5 | 4 | 4 | 4 | 5 | 5 | 5 | 5 | 5 | 5 |
| ZC1 | 5 | 5 | 5 | 5 | 5 | 3 | 5 | 5 | 5 | 5 | 4 | 3 | 4 | 4 | 5 | 4 | 5 | 4 | 3 | 2 | 4 | 5 | 5 | 3 | 5 | 3 | 3 | 4 | 4 | 3 | 4 | 5 | 4 | 3 | 2 | 3 | 3 | 4 | 4 | 3 |
| ZC2 | 5 | 5 | 5 | 5 | 5 | 5 | 5 | 5 | 5 | 5 | 5 | 3 | 4 | 4 | 5 | 4 | 3 | 4 | 3 | 4 | 4 | 5 | 5 | 4 | 5 | 5 | 4 | 3 | 5 | 5 | 5 | 4 | 3 | 4 | 5 | 3 | 4 | 3 | 5 | 3 |
| ZC3 | 5 | 5 | 4 | 4 | 3 | 3 | 5 | 5 | 4 | 3 | 5 | 4 | 3 | 4 | 5 | 3 | 4 | 2 | 5 | 2 | 3 | 5 | 4 | 2 | 5 | 4 | 5 | 3 | 5 | 4 | 4 | 3 | 4 | 4 | 4 | 3 | 3 | 5 | 5 | 2 |
